# An AI-driven approach for modeling the compressive strength of sustainable concrete incorporating waste marble as an industrial by-product

**DOI:** 10.1038/s41598-024-77908-3

**Published:** 2024-11-05

**Authors:** Ramin Kazemi, Seyedali Mirjalili

**Affiliations:** 1 Independent Researcher, Sabzevar, Iran; 2 Centre for Artificial Intelligence Research and Optimisation (AIRO), Torrens University Australia, Brisbane, Australia; 3 University Research and Innovation Center, Obuda University, Budapest, Hungary; 4 Department of Computer Science, VSB-Technical University of Ostrava, Ostrava, Czech Republic

**Keywords:** By-product, Environmental policies, Waste marble, Sustainable concrete, Compressive strength, Artificial intelligence, Civil engineering, Materials science, Mathematics and computing

## Abstract

A key goal of environmental policies and circular economy strategies in the construction sector is to convert demolition and industrial wastes into reusable materials. As an industrial by-product, Waste marble (WM), has the potential to replace cement and fine aggregate in concrete which helps with saving natural resources and reducing environmental harm. While many studies have so far investigated the effect of WM on compressive strength (CS), it is undeniable that conducting experimental activities requires time, money, and re-testing with changing materials and conditions. Hence, this study seeks to move from traditional experimental approaches towards artificial intelligence-driven approaches by developing three models—artificial neural network (ANN) and hybrid ANN with ant colony optimization (ACO) and biogeography-based optimization (BBO) to predict the CS of WM concrete. For this purpose, a comprehensive dataset including 1135 data records is employed from the literature. The models’ performance is assessed using statistical metrics and error histograms, and a *K*-fold cross-validation analysis is applied to avoid overfitting problems, emphasize the models’ reliable predictive capabilities, and generalize them. The statistical metrics indicated that the ANN-BBO model performed best with a correlation coefficient (R) of 0.9950 and root mean squared error (RMSE) of 1.2017 MPa. Besides, the error distribution results revealed that the ANN-BBO outperformed the ANN and ANN-ACO with a narrower range of errors so that 98% of the predicted data points in the training phase by the ANN-BBO model experienced errors in the range of [-10%, 10%], whereas for the ANN-ACO and ANN models, this percentage was 85% and 79%, respectively. Additionally, the study employed SHapley Additive exPlanations (SHAP) analysis to clarify the impact of input variables on prediction accuracy and found that the specimen’s age is the most influential variable. Eventually, to validate the ANN-BBO, a comparison was performed with the results of previous studies’ models.

**Supplementary Information:**

The online version contains supplementary material available at 10.1038/s41598-024-77908-3.

## Introduction

### Background and literature review

**Fig. 1:**
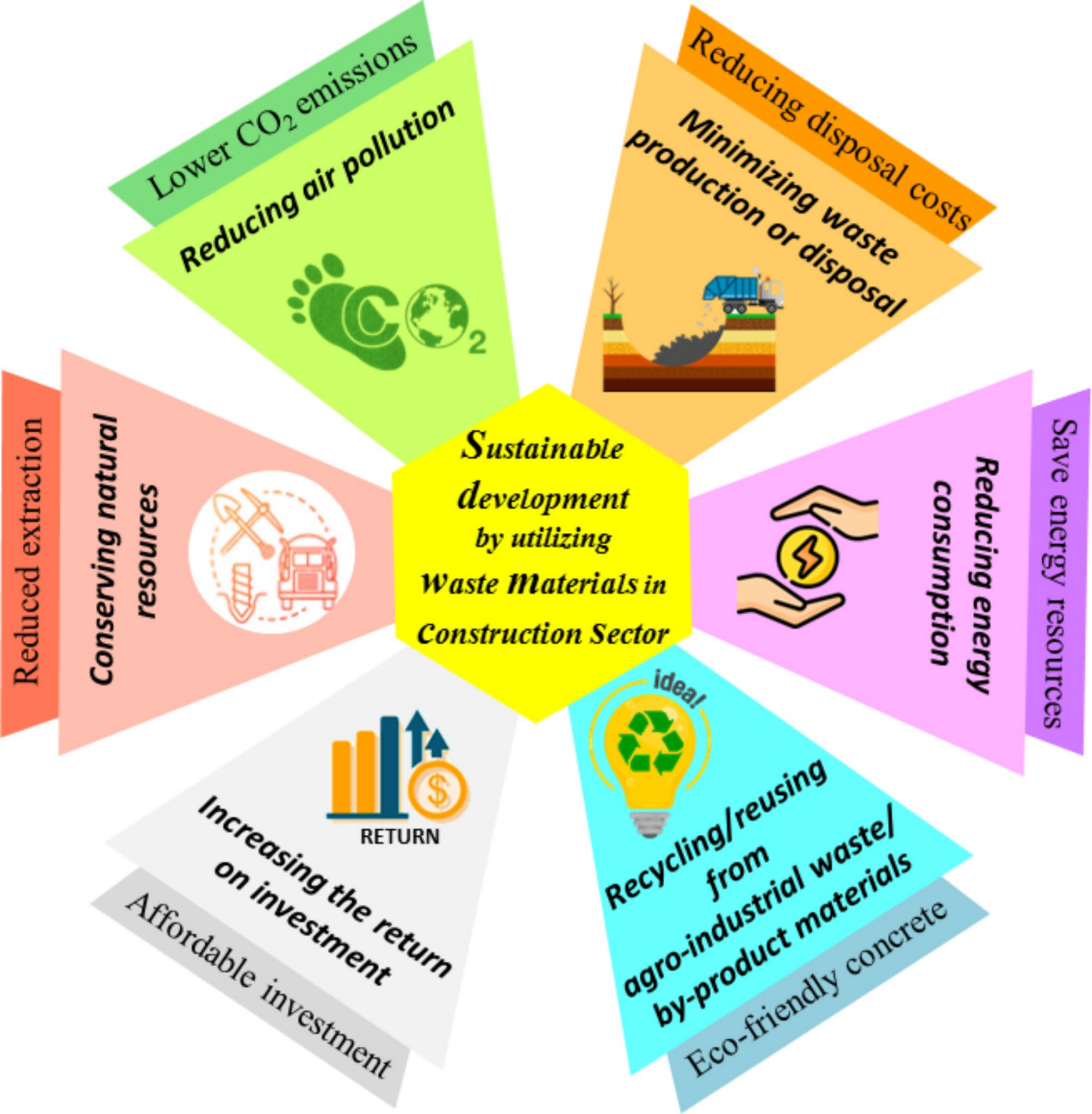
The effect of using by-products and waste materials in the sustainable construction sector.

Dealing with the category of environmental sustainability and preservation is one of the priorities of today’s world on which global communities agree. In this regard, the concrete industry is taking steps towards eco-friendly construction by replacing agro-industrial by-products and wastes instead of cement and natural aggregate sources^1^. This issue is important from two aspects: (i) it is undeniable that with the continuation of cement demand and natural resources exploitation, there will be irreparable consequences resulting from high energy consumption, environmental threats, and tampering with nature; and (ii) ignoring the large amount of waste and releasing or burying it in nature is the source of environmental hazards besides insufficient landfills and high disposal costs. Figure 1 illustrates the effect of using by-products and waste materials in the sustainable construction sector.

Waste marble (WM) is an industrial by-product that is obtained during various processes of cutting, shaping, and polishing marble. Its disposal in the ecosystem leads to environmental problems, especially in the soil, including reduced porosity and permeability and endangering fertility^2^. On the other hand, the WM, due to having fine particles and high lime content, can improve concrete’s mechanical properties and durability through two effects of micro-filler and nucleation^3^. This means that the WM catalyzes the hydration process by creating preferential nucleation sites and facilitating the surface interaction of filler grains and calcium ions with the formation of calcium silicate hydrate, thereby strengthening the hardened cement paste^4^. Furthermore, the ability to substitute in both components of concrete materials, i.e. cement and fine aggregate, is another unique feature of the WM, distinguishing it from other substitute materials.

Before large-scale utilization, it is essential to carefully evaluate the effect of WM in WM-containing concretes (WMC) on the compressive strength (CS) as one of the most crucial design criteria in concrete structures. In recent years, many experimental researches have been conducted to determine the optimal percentage of WM in concrete as a partial substitution for cement and fine aggregate with the aim of reducing cement consumption and conserving natural aggregate sources^5–8^. By scrutinizing the available studies, it was found that the optimal percentage of replacement instead of cement was reported as 5%^9–11^, 10%^3,12–15^, and 15%^16^, with more references to 10%. In the optimal content, owing to its microstructure, waste marble fills the small pores and improves the mechanical performance of concrete because of its calcium silicate hydrate reaction^3,15^. On the other hand, in high waste marble content, the dilution effect leads to less hydrated compounds due to the less clinker content of the blended cement. This means that it induces the potential reduction in the cementing materials, which is commonly known as the dilution of the pozzolanic reactions^17,18^. For fine grains, various optimal replacement percentages of 10%^4,19^, 15%^20^, 20%^21^, and 40%^6–8^ were recommended. Although adopting the traditional laboratory-based method to determine the CS of WMC can provide accurate evaluations, it is faced with challenges including: (i) its time-consuming and expensive nature, (ii) the need for re-testing with changes in the material proportions or testing conditions, and (iii) lack of comprehensiveness arising from the limited number of prepared mixtures and tested samples. In light of this, an alternative and effective solution is to adopt an artificial intelligence (AI)-driven approach to overcome the aforementioned challenges and minimize the dependence on traditional laboratory methods while achieving reliable results.

During the last decade, many researchers have turned to using AI-driven techniques to estimate the mechanical properties of concrete^22–29^. Table 1 concisely lists recent AI-driven studies focused on predicting the CS of sustainable concretes containing agro-industrial by-products and wastes.

**Table 1:** Overview of AI-driven studies to estimate the CS of concretes containing agro-industrial by-products and wastes.

Type of by-products and wastes used in concrete	No. of data used	AI technique	The best-proposed model (*R* value)	Authors [Ref.]
Fly ash and silica fume	85	• ANN • SVM	SVM (0.9905)	Abunassar et al.^30^
Seashell	202	• ANN • MARS • M5P	ANN (0.9889)	Alidoust et al.^31^
Waste foundry sand	340	• ANN-PSO • ANN-ACO	ANN-ACO (0.9971)	Kazemi et al.^32^
Ground granulated blast furnace slag	625	• DT • RF • SVM • KNN	DT (0.9700)	Kioumarsi et al.^33^
Sugarcane bagasse ash	340	• SVM • RBF • ANN	SVM (0.9900)	Pazouki et al.^34^
Waste marble powder	158	• ANN • RF • Gaussian process • SVM • ANFIS • RT	SVM (0.9415)	Sharma et al.^35^
Rice husk ash	909	• ANN • MARS • M5P	ANN (0.9889)	Tavana Amlashi et al.^36^
Waste glass	830	• MLP-MPA • ANFIS-MPA • SVR-MPA • LSSVR-MPA	LSSVR-MPA (0.9915)	Ben Seghier et al.^37^
Ceramic waste	45	• DT • AdB • RF • Bg • GB • XGB	RF (0.9845)	Chang et al.^38^
Electronic waste	279	• MEP	MEP (0.944)	Shah et al.^39^

Various AI-driven predictive techniques have been known so far, among which the artificial neural network (ANN) method has been identified as a robust tool because of its ability to: (i) identify patterns, (ii) establish an effective relationship between inputs and output, (iii) model non-linear statistical data, (iv) tolerate errors, and (v) flex to solve complex real-world issues^40^. Based on this, the utilization of ANN to predict the properties of eco-friendly concretes has received more attention (e.g.,^41–43^).

A thorough literature review shows that until now, only a few AI studies have been developed to predict the CS of WMC^35,44–48^. Table 2 provides a detailed list of relevant previous efforts.

**Table 2:** A summary of previous research on the applications of AI techniques to model the CS of WMC.

Authors [Ref.]	Year	No. of data used	Input variables	The best proposed model	Accuracy on the best model (*R*^2^ value)
Sharma et al.^48^	2022	49	*5* variables: Cement-Fine aggregate-Coarse aggregate-Water/Cement-Waste marble	ANN	0.9805
Sharma et al.^47^	2022	189	*6* variables: Cement-Fine aggregate-Coarse aggregate-Water-Waste marble-Age	GP	0.8410
Singh et al.^45^	2022	720	*7* variables: Cement-Fine aggregate-Coarse aggregate-Water-Slump-Super plasticize-Waste marble	RF	0.9260
Sharma et al.^35^	2023	158	*6* variables: Cement-Fine aggregate-Coarse aggregate-Water-Waste marble-Age	RT	0.9551
Shamsabadi et al.^44^	2023	630	*7* variables: Cement-Fine aggregate-Coarse aggregate-Water-Waste marble-Super plasticize-Age	XGB	0.9800
Sharma et al.^46^	2023	130	*6* variables: Cement-Fine aggregate-Coarse aggregate-Water/Cement-Waste marble-Age	ANFIS	0.9944

A closer look at these studies brings to mind several points. First and foremost, the dataset used by researchers to present their AI models has been limited in most cases. Especially with a closer examination, it is clear that the highest accuracy belongs to the models with the least number of data; for example, R^2^ value = 0.9944 for^46^ and R^2^ value = 0.9805 for^48^. This means that achieving these models with high accuracy cannot indicate their strong performance, while their performance against a wider database is still questionable. Besides, all the proposed models are single models, while despite the superiority of hybrid models with meta-heuristic optimization techniques, they have not been utilized yet.

Recently, the development of AI models has been influenced by the use of metaheuristic techniques in the form of combination models with predictive models so that this hybrid approach maximizes the strengths of each single-mode prediction model and minimizes its defects through discovering efficient solutions and improving the accuracy^49–53^. Consequently, it induces more reliable outcomes.

### Research gaps

Scrutinizing the literature background indicated that there are still gaps that need to be tackled to achieve more precise and reliable AI models. Specifically, the gaps extracted from the literature that this study pursuits to fulfill include the following:

- Considering the superiority of hybrid AI models, the best-proposed models by the literature so far were single-mode models.
- Previous models were often developed based on a limited number of data records related to the CS of WMC. It is undeniable that the utilization of a more comprehensive database leads to the achievement of more accurate and reliable models.
- Previous studies were only satisfied with presenting their model without comparing results with preceding models and evaluating the validity of their model, which is known as the last essential step in model development.

Undoubtedly, it calls for the need to address these gaps.

### Research objectives

This study deals with sustainable concrete incorporating WM, and this by-product is a serious concern in the marble industry due to both disposal and environmental problems. Based on this, the ability to partially replace WM instead of cement and fine aggregate in concrete is an effective solution to tackle the mentioned concerns and conserve natural resources. Undoubtedly, traditional laboratory-based methods require time and money, as well as re-testing with changing materials and conditions. Hence, the time has come to adopt an AI-driven technique aiming to provide an alternative strategy and minimize the reliance on laboratory activities. To address the gaps in the previously mentioned AI studies regarding predicting the CS of WMC, the present study seeks the following main objectives:

- Develop two hybrid AI-driven techniques by integrating metaheuristic optimization algorithms-ant colony optimization (ACO) and biogeography-based optimization (BBO) with ANN to predict the CS of WMC.
- Utilize an extensive database comprising 1135 data records taken from 53 literature sources.
- Present a comprehensive SHapley Additive exPlanations (SHAP) analysis in order to enrich the discourse by providing in-depth insights into the influencing variables on CS of WMC, a perspective not thoroughly investigated in existing literature.
- Establish a comparative analysis to validate the performance of the best-proposed model in the current study compared to literature models.

**Fig. 2:**
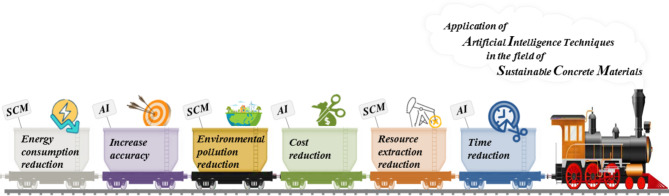
Footprints of artificial intelligence and sustainable concrete materials as a research roadmap in the current research.

At a glance, Fig. 2 illustrates the footprints of the AI-driven approach and sustainable concrete materials as a research roadmap in this study.

The following is how the rest of the paper is structured: (i) an overview of the AI-driven techniques utilized; (ii) a description of the database used; (iii) an outline of the process of models’ construction and evaluation; (iv) an evaluation and discussion of the results in four aspects: (1) tuning hyperparameters; (2) evaluating models’ performance; (3) analyzing the contribution of input variables; and (4) validating the best-proposed model by comparing with literature models; and finally, (v) a summary of the key findings and future directions.

## Methodological background

### Artificial neural network (ANN)

**Fig. 3:**
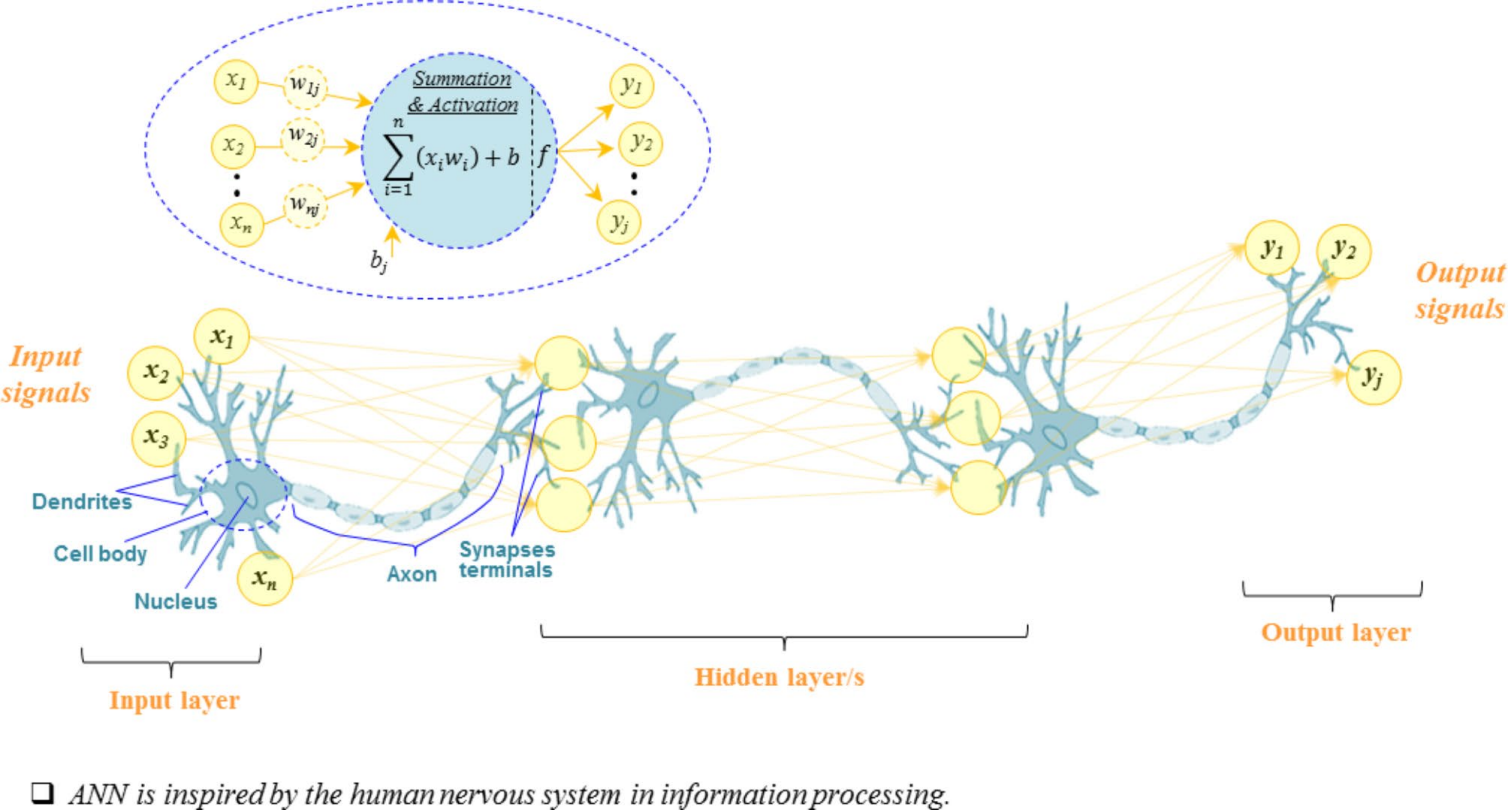
A typical multi-layer neural network model architecture.

ANNs represent a widely employed method in the field of AI, known for their high performance in predictive modeling^54^. Inspired by the human neural system, the input, hidden and output layers form the main framework of the ANN model. The input layer, representing independent variables (input signals), constitutes the input data to the model. The output layer, equivalent to the independent data, is then extracted. Hidden layers uncover the relationship between the two previously mentioned layers. The selection of optimal input signals contributes to obtaining a more accurate model. Figure 3 depicts a schematic of a neural network with multiple hidden layers. In this approach, each input signal can be assigned a specific weight, considering its impact on the output signal. The output signal is obtained using the following Eq:

1 $${Output}_{j}=f\left(\sum_{i=1}^{n}{{Input}_{i} \times weight}_{ij}+ {bias}_{j}\right)$$

where *f* is the activation function and *n* is the number of input signals.

### Ant colony optimization (ACO)

The ACO method, an optimization technique inspired by the foraging behaviour of ants in search of food, was first introduced in 1992. Blind ants, as they move toward food, emit a substance called pheromone along their path. This pheromone allows other ants to identify the shortest path by sensing the concentrations on different routes. The greater the concentration of pheromones on the way, the shorter the route toward the food^55^. The ACO algorithm can be defined as follows:

2 $$Probability_{{ij}} = \left\{ {\begin{array}{*{20}l} {\frac{{\left[ {\tau _{{ij}} } \right]^{\alpha } \left[ {\eta _{{ij}} } \right]^{\beta } }}{{\sum\limits_{{l \in N_{i} }} {\left[ {\tau _{{ij}} } \right]^{\alpha } } \left[ {\eta _{{ij}} } \right]^{\beta } }},} \hfill & {if\,j \in N_{i} } \hfill \\ {0,} \hfill & {otherwise} \hfill \\ \end{array} } \right.$$

where *τ*_*ij*_ and *η*_*ij*_ = Pheromone and heuristic information, *α* and *β* = Relative importance, positive constants.

By finding the destination route by ants, the pheromone deposited on the edges is obtained from the following Eqs. [55]:

3 $${\tau }_{ij}=\left(1-\rho \right){\tau }_{ij}+\Delta {\tau }_{ij}$$

4 $$\Delta {\tau }_{ij}= \sum_{z=1}^{m}\Delta {{\tau }_{ij}}^{z}$$

5 $$\Delta \tau _{{ij}}^{z} = \left\{ {\begin{array}{*{20}l} {\frac{1}{{L_{z} }}~,} \hfill & {if\,\left( {i,~j} \right) \in ~E_{z} } \hfill \\ {0,} \hfill & {otherwise} \hfill \\ \end{array} } \right.$$

where:

*ρ* = Pheromone evaporation coefficient, *m* = No. of ants, ${\Delta \tau }_{i,j}^{z}$ = Pheromones deposited by ant *z*, *L*_*z*_ = Path length by ant *z*, *E*_*z*_ = Edges by ant *z*. *E*_*z*_ = Edges by ant *z*.

**Fig. 4:**
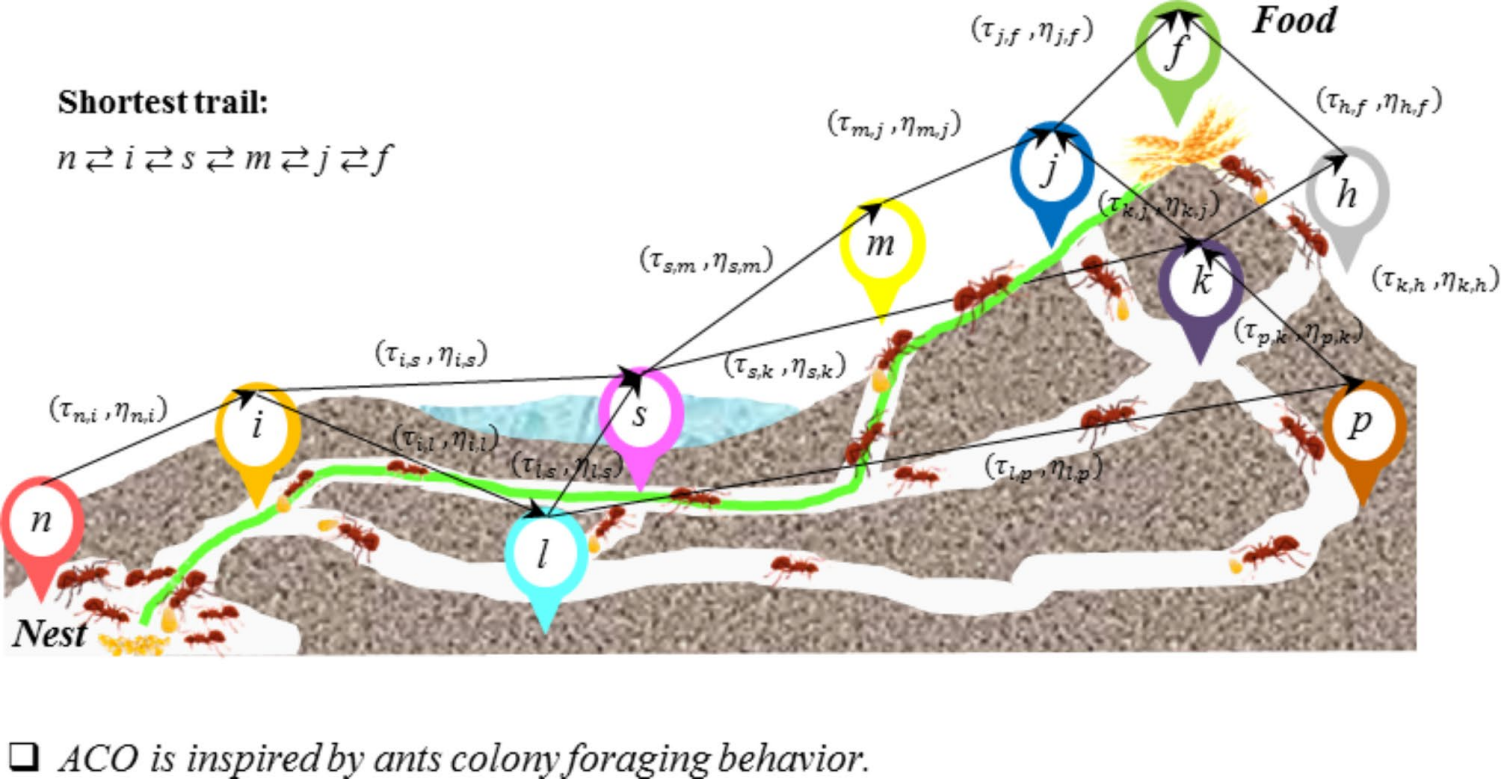
The schematic performance of the ACO method.

Figure 4 shows the schematic performance of the ACO method.

### Biogeography-based optimization (BBO)

**Fig. 5:**
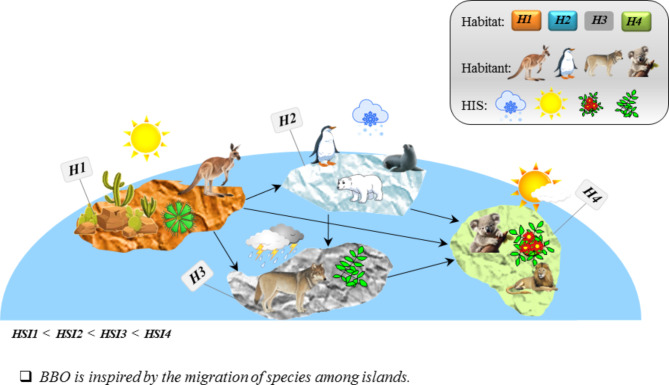
The schematic performance of the BBO method.

The BBO method, introduced by Simon in 2008, is a nature-inspired optimization algorithm that examines the distribution of species across geographical landscapes. Developed as a heuristic optimization technique, BBO replicates the processes of migration, mutation, and natural selection observed in biological ecosystems to find optimal solutions to complex problems^56^. The principles of BBO are rooted in the concept of migration. BBO employs the idea of migration by representing potential solutions as candidate solutions or habitats. These solutions undergo continuous adjustments and information exchanges to enhance the population’s quality over successive iterations. Migration in BBO is facilitated through the exchange of information, or migration vectors, between different habitats or candidate solutions. The probability of a solution migrating to a new habitat is influenced by suitability index variables (SIV) and habitat suitability index (HSI), reflecting independent variables and the homeland’s suitability, respectively^57^. Mutation is another key principle in BBO, reflecting the adaptability observed in biological systems. The algorithm introduces diversity into the population through random changes in solutions, preventing premature convergence to suboptimal solutions. The mutation process enables BBO to explore a broader solution space and discover potentially better solutions. This method evaluates solutions based on their fitness, representing their ability to solve the given problem. Solutions with higher fitness are more likely to be selected for reproduction, while those with lower fitness may be eliminated. This process emulates the survival of the fittest and drives the population toward improved solutions over time. The algorithm iteratively refines the population, leading to improved solutions. Figure 5 illustrates the schematic performance of the BBO method.

The principal formulations of the BBO method are as follows:

6 $${I}_{s}= {I}_{max}(1-\frac{s}{{s}_{max}})$$

7 $${E}_{s}={E}_{max}\frac{s}{{s}_{max}}$$

8 $${M}_{s}={M}_{max}(1-\frac{{P}_{s}}{{P}_{max}})$$

where *s* and *s*_*max*_ = Number and maximum of habitats’ species, *I* and *E* = immigration and emigration, *M* and *P* = Mutation and probability.

It is not without merit, investigating the strengths of the optimization techniques-ACO and BBO used in this study can clarify the justification of their superiority compared to other techniques. The advantage of ACO is significant in several aspects: (i) Strength: even if some ants fail, the exploration continues towards the optimal path; and (ii) Self-organization: achieving solution paths are reinforced during the exploration process rather than pre-defined^58–60^. A highly cited study^61^ evaluated BBO’s performance using five well-known heuristic methods. The results indicated that BBO has the ability to make abrupt changes in candidate solutions due to migration and crossover strategies. This unique feature distinguishes it from other methods that often experience the problem of getting trapped in local minima. In addition, the lack of need for derivatives of the objective function due to its stochastic nature makes it efficient to overcome a wide range of real-world problems^62,63^.

Hence, this study, for the first time, seeks to use these AI techniques in the form of combination models to predict the CS of WMC.

## Data description

This study collected an extensive dataset including 1135 different mixtures to predict the CS of WMC from 53 literature sources^3,5–11,15,16,18–21,64–102^. The complete details of all mixtures can be found in the Supplementary File. In the constructed models, the amounts of cement (C), water (W), waste marble (WM), fine aggregate (FA), coarse aggregate (CA), superplasticizer (SP), and age of specimens (A) are considered as inputs (independent variables), and the CS is considered as the output (dependent variable). It is important to note that it was not possible to consider other characteristics of waste marble because a limited number of studies have introduced the details in this regard, so there was no access to a comprehensive database, which is an essential need for AI models.

**Fig. 6:**
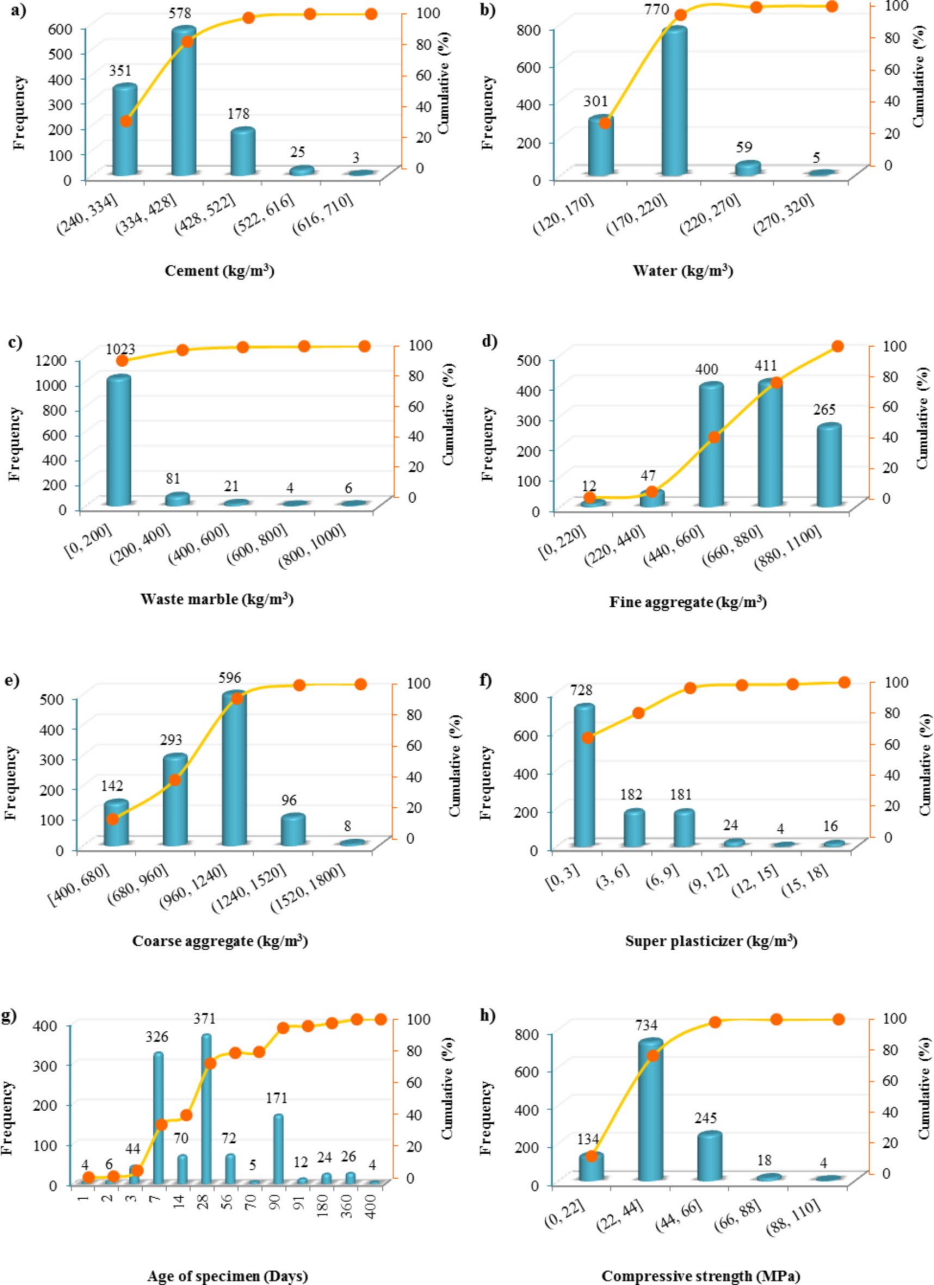
Frequency histograms of variables.

The frequency of inputs and output is shown in Fig. 6. As illustrated in this figure, about 80% of mixtures have the content of cement, water, and WM in the range of (240, 428] kg/m^3^, (120, 220] kg/m^3^, and [0, 200] kg/m^3^, respectively. Also, the most frequent ages of the specimens are 28, 7, and 90 days. The range of CS in about 70% of specimens is between 22 and 24 MPa. The descriptive statistics of the inputs and output, including total range, mean, standard deviation, Kurtosis, and Skewness, are provided in Table 3.

**Table 3:** Descriptive statistics of the variables.

Variable (Acronym)	Type of variable	Unit	Range	Mean	Standard deviation	Kurtosis	Skewness
Cement (C)	Input	kg/m^3^	245.00-708.80	373.152	67.731	0.795	0.783
Water (W)	Input	kg/m^3^	125.30-306.52	184.794	24.874	1.192	0.447
Waste marble (WM)	Input	kg/m^3^	0-972.00	88.444	114.520	17.334	3.431
Fine aggregate (FA)	Input	kg/m^3^	0-1095.00	732.824	182.057	0.858	-0.436
Coarse aggregate (CA)	Input	kg/m^3^	421.00-1750.97	1001.214	226.039	-0.709	-0.307
Super plasticizer (SP)	Input	kg/m^3^	0-16.20	2.849	3.484	1.412	1.275
Age of specimen (A)	Input	Days	1-400	44.002	63.975	14.604	3.557
Compressive strength (CS)	Output	MPa	1.50-100.50	36.457	12.932	1.829	0.897

**Fig. 7:**
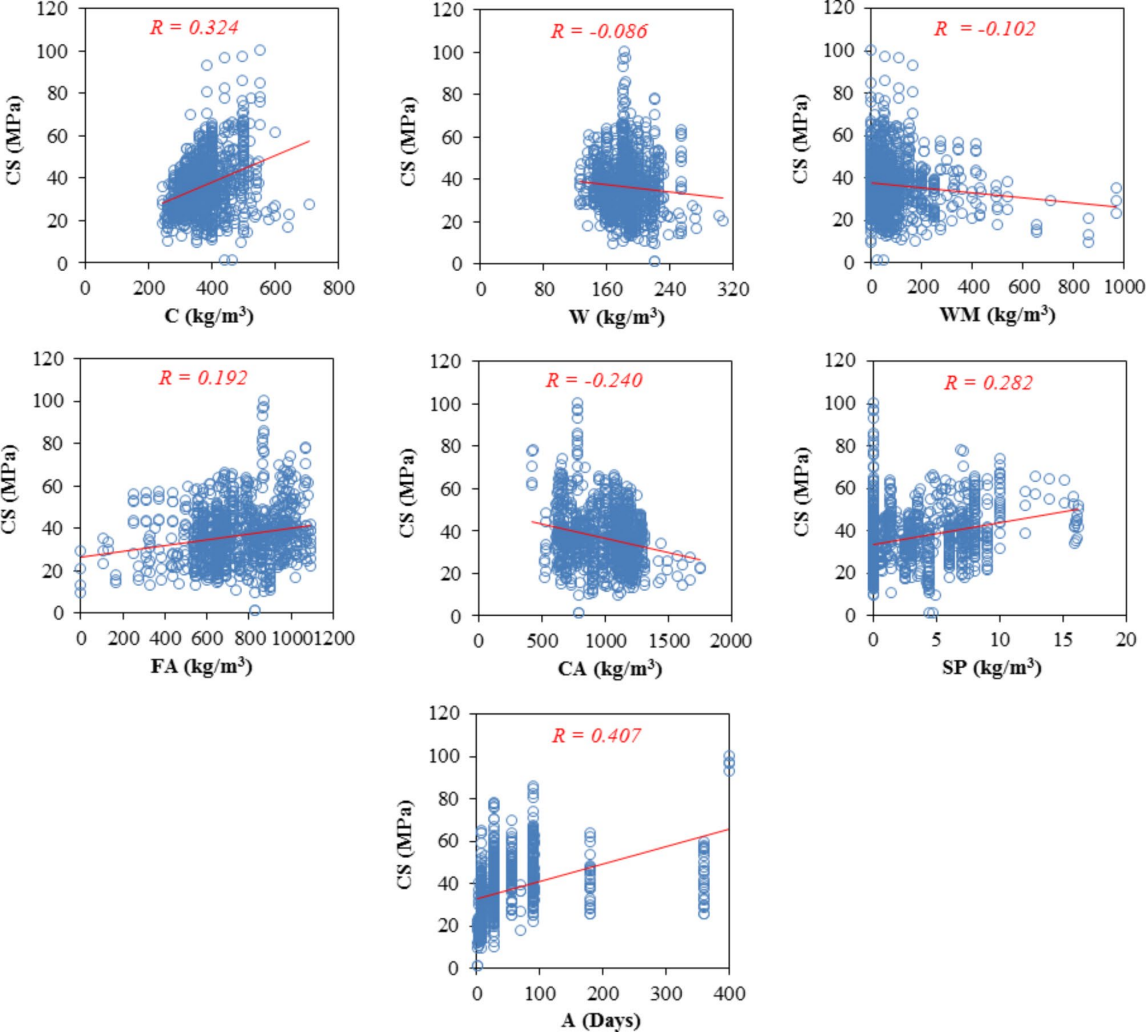
Scatter plots of predictor variables vs. the target variable.

**Fig. 8:**
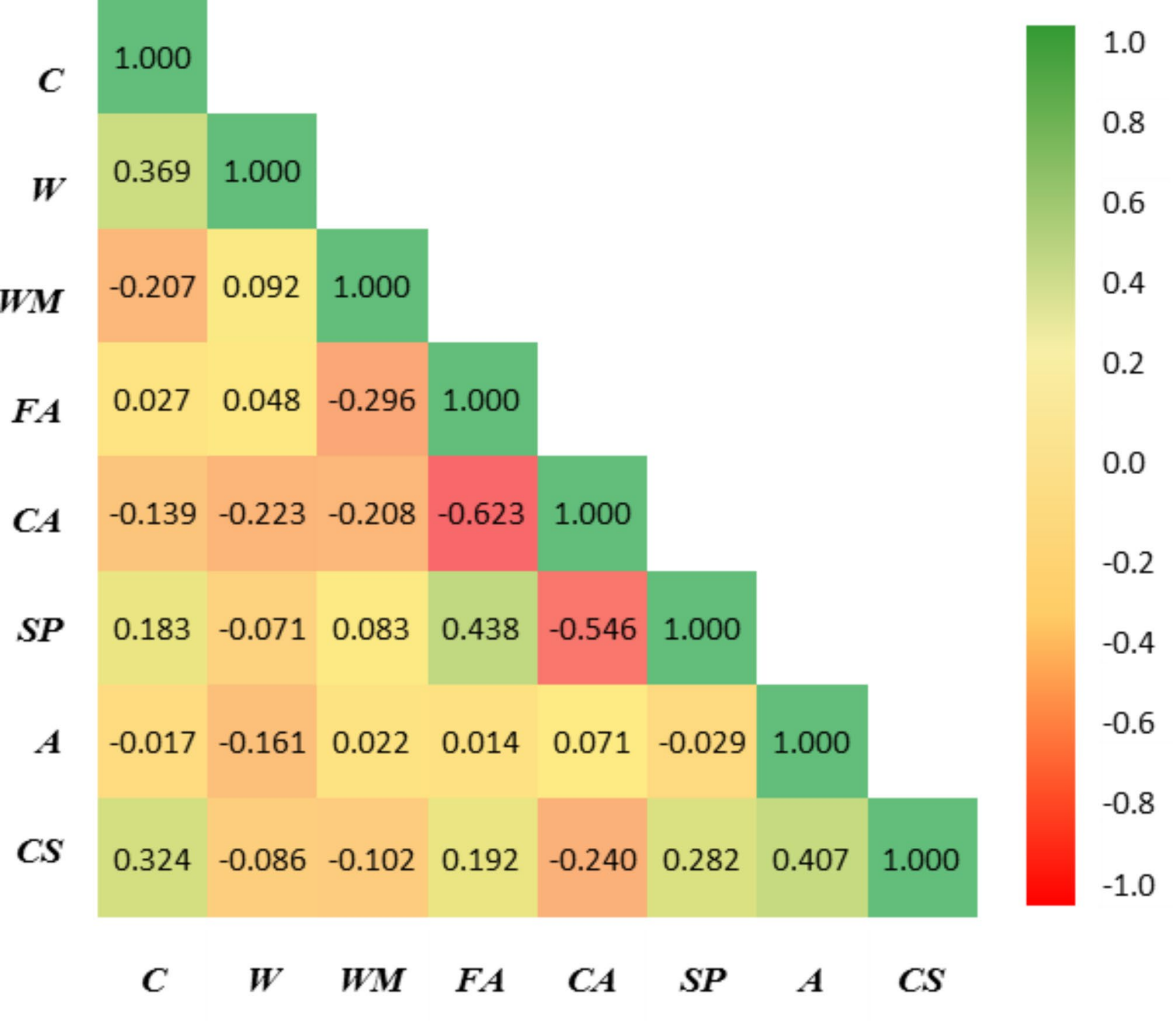
Pearson’s correlation coefficient between input and output variables.

The accuracy of predictive models highly relies on choosing the input as an independent variable to predict the output. AI methods try to find a relation between these variables, so choosing appropriate and effective variables helps to achieve more accurate models. The correlations between the variables are explored to discover the appropriateness of each characteristic variable. To investigate the relationship between the characteristic variables, the Pearson correlation coefficient approach is applied. Indeed, Pearson’s correlation coefficient is a statistical measure that evaluates the strength and direction of the relationship between two variables. The purpose of Figs. 7 and 8 is to highlight the distribution of pairwise correlation between variables. In this regard, a sensitivity analysis based on linear correlation between inputs and output (CS) has been performed. As illustrated in Fig. 7, the age of specimens has the strongest correlation with CS, followed by cement. However, the water content indicates a weak linear relationship with CS, contrary to common observations in the concrete technology literature. This is because of the fact that this relationship for the water variable does not follow a linear pattern that Pearson’s coefficient can capture effectively. Furthermore, considering that the range of water consumption in the concrete mix design is typically within a narrow range of 150–220 kg/m^3^, the ability to detect a linear relationship is reduced, leading to a lower correlation. In summary, a low correlation coefficient between the water variable and CS can be justified by the narrow data range and the presence of a non-linear association.

Figure 8 shows Pearson’s correlation coefficient between variables, which is in agreement with linear correlation; the age of the specimens shows the most correlation with CS. However, Pearson’s correlation shows a poor linear relation between variables since there is a nonlinear and complex relation between them. This demonstrates that there is a need for more complicated tools like AI to find this relation and predict the CS of WMC with appropriate accuracy.

## Model construction and evaluation

### Developed models

This study seeks to utilize an AI-driven approach to model the CS of WMC. To this end, three AI models-ANN, ANN-ACO, and ANN-BBO have been proposed. It could provide beneficial insight into the performance of the single ANN model and its combination models. The application of two techniques-ACO and BBO is to figure out the optimum variables in such a way that the predictive model is directed towards improving more accurate results with minimum error. In this regard, the first step is to randomly divide the entire dataset (1135 data point) into three phases: training_*phase*_, validation_*phase*_, and testing_*phase*_, with proportions of 70% (795), 15% (170), and 15% (170), respectively. The purpose of these divided phases is to train the model (training_*phase*_), to avoid overfitting (validation_*phase*_), and to verify the model after implementation (testing_*phase*_). The next step is the normalization of the data. This process significantly affects the development of the model, as it converts variables with different ranges into unitless variables in a certain range and diminishes the effect of higher values on lower values^103^. Equation (9) was used for data normalization into the range of [-1, 1], where *P*_*i*_ represents either an input or an output variable.

9 $${P}_{ normalized}=\frac{2\left({P}_{i}-{P}_{minmum}\right)}{{P}_{maximum}-{P}_{minmum}}-1$$

Many studies conducted regarding the evaluation of different learning algorithms indicated the superior performance of the Levenberg-Marquardt algorithm^32,104,105^. Hence, this algorithm is considered for this study. In addition, regarding the transfer function, the hyperbolic tangent function is chosen according to the recommendations of several studies^106–108^. The number of hidden layer/s and their nodes in the architectural structure of the model affects the convergence of the model learning^109^. Following the universal approximation theorem^110^, a single hidden layer is assigned for models. Thus, the initial architectural structure of the model is *7*-*H*_*nodes*_-*1*. The number of *H*_*nodes*_ (i.e. No. nodes in the hidden layer) is related to the number of inputs and output^111^. To specify the final architectural structure of the model (i.e. *H*_*nodes*_), a detailed analysis will be performed in subsection 5.1.

### Evaluation indicators

Some indicators and errors, such as MAE, RMSE, RRMSE, R^2^, NSE, PI, and A10-index, have been calculated to evaluate the accuracy of the proposed models. Their equations and ranges are provided in Table 4.

**Table 4:** The statistical measures. [T = Total of data. $\:{CS}_{i,\:obs}$ and $\:{CS}_{i,\:pre}$ = The observed and predicted *CS* of the *i*th data, respectively. $\:\stackrel{-}{{CS}_{obs}}$ and $\:\stackrel{-}{{CS}_{pre}}$ = The average of the observed and predicted *CS*, respectively. m10 = The number of data in which their $\:{CS}_{obs}$/$\:{CS}_{pre}$ ratio fits in the range of 0.90–1.10.]

Indicator	Equation	Range ideal
Mean absolute error (MAE)	$\:\frac{1}{\text{T}}\sum\:_{\text{i}=1}^{\text{T}}\left|{CS}_{i,\:obs}-{CS}_{i,\:pre}\right|$	(0, $\:+\infty\:$) 0
Root mean squared error (RMSE)	$\:\sqrt{\frac{1}{\text{T}}\sum\:_{\text{i}=1}^{\text{T}}{({CS}_{i,\:obs}-{CS}_{i,\:pre})}^{2}}$	(0, $\:+\infty\:$) 0
Relative root mean squared error (RRMSE)	$\:\frac{\text{R}\text{M}\text{S}\text{E}}{\stackrel{-}{{CS}_{obs}}}$	(Excellent < 0.05) (0.05 < Good < 0.1) (0.1 < Fair < 0.15) (Poor > 0.15)
Coefficient of determination (R^2^)	$\left( {\frac{{\sum\nolimits_{{{\text{i}} = 1}}^{{\text{T}}} {\left( {CS_{{i,\:obs}} - \mathop {CS_{{obs}} }\limits^{ - } } \right)\left( {CS_{{i,\:pre}} - \mathop {CS_{{pre}} }\limits^{ - } } \right)} }}{{\sqrt {\left[ {\sum\nolimits_{{{\text{i}} = 1}}^{{\text{T}}} {\left( {CS_{{i,\:obs}} - \mathop {CS_{{obs}} }\limits^{ - } } \right)^{2} } } \right]\left[ {\sum\nolimits_{{{\text{i}} = 1}}^{{\text{T}}} {\left( {CS_{{i,\:pre}} - \mathop {CS_{{pre}} }\limits^{ - } } \right)^{2} } } \right]} }}} \right)^{2}$	(0, 1) 1
Nash-Sutcliffe efficiency (NSE)	$1 - \frac{{\sum\nolimits_{{{\text{i}} = 1}}^{{\text{T}}} {\left( {CS_{{i,obs}} - CS_{{i,pre}} } \right)^{2} } }}{{\sum\nolimits_{{{\text{i}} = 1}}^{{\text{T}}} {\left( {CS_{{i,obs}} - \mathop {CS_{{obs}} }\limits^{ - } } \right)^{2} } }}$	($\:-\infty\:$, 1) 1
Performance index (PI)	$\:\frac{\text{R}\text{R}\text{M}\text{S}\text{E}}{1+\text{R}}$	(0, 1) 0
A10-index	$\:\frac{\text{m}10}{\text{T}}$	(0, 1) 1

### K-fold cross-validation

**Fig. 9:**
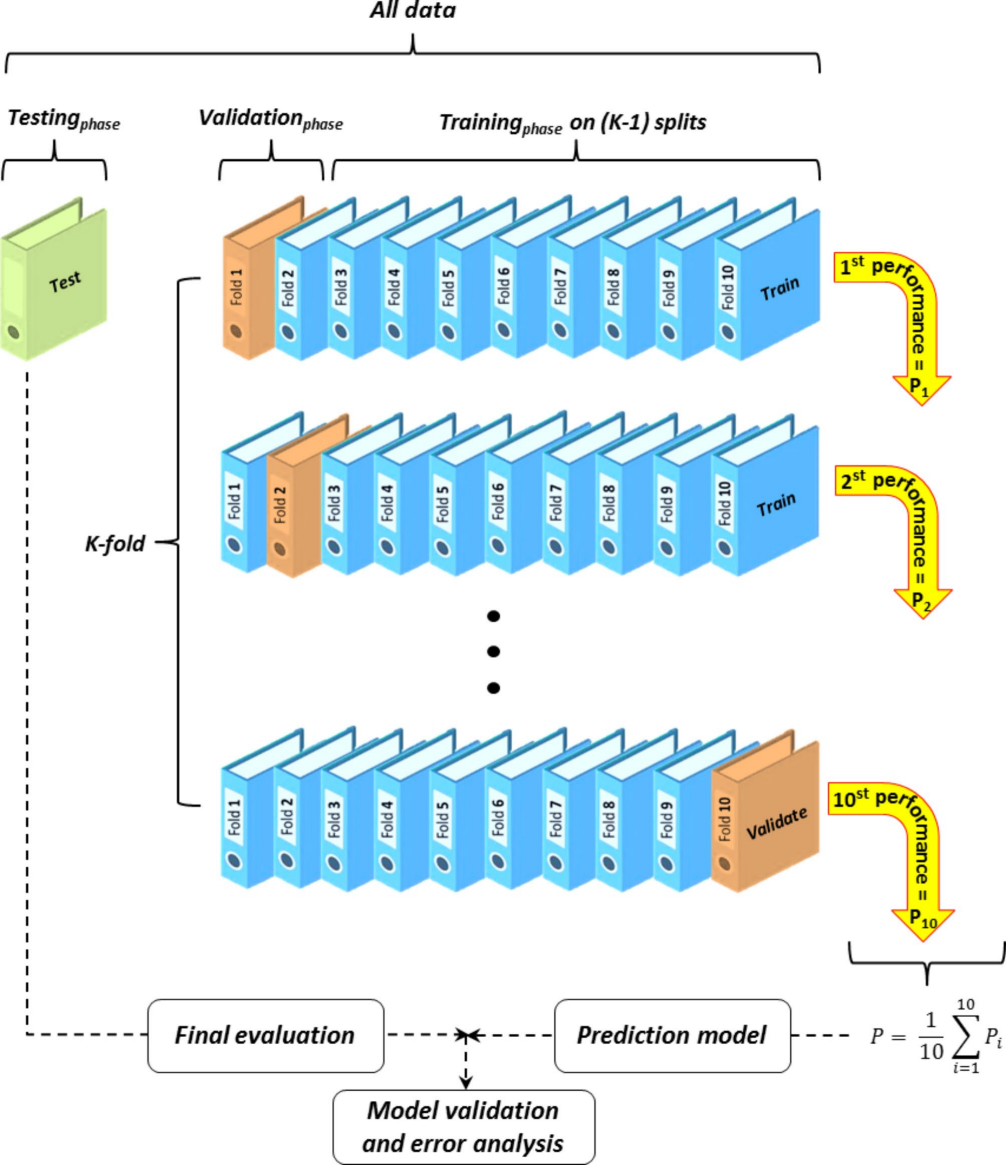
10-fold cross-validation method for data separation and validating models.

For the purpose of training, validation, and testing the proposed models, data collected, consisting of 1135 composite designs, has been subjected to *K*-fold cross-validation. The *K*-fold cross-validation is a common analysis for evaluating the performance of predictive models, where the data is divided into *K* groups^112^. The model is then trained on *K*-1 groups and validated on the remaining group. This process is repeated until each of the *K* groups has been used as the validation data, and the average performance of the model is calculated over each iteration^113^. In this research, the data designated for the training_*phase*_ and validation_*phase*_ are randomly partitioned into ten folds, where 90% of the data in each fold is used for training, and 10% is used for validation. The error for each fold is calculated, and the average is computed. Subsequently, the predictive models obtained are tested in a separate testing_*phase*_ to assess the overall model performance and undergo re-validation. The process is shown in Fig. 9. This approach enhances the credibility of the presented model and reduces the error rate in modelling by systematically validating its performance on distinct subsets of the data.

### Computational time complexity of the proposed AI models

By analyzing the computational complexity as one of the performance evaluation criteria, understanding the real computational cost of the proposed models is facilitated. To describe this feature, the big-O notation is utilized to represent the efficiency of a model in the worst case with respect to its input dimension^114^. For this purpose, the total complexity of the used models can be obtained by adding each complexity for all stages. To begin, For an ANN, the complexity depends on the number of *i*, *h*, and *o*, which respectively refer to input, hidden, and output neurons. The number of data involved in the train_*phase*_ and epochs are denoted by *d*_*t*_ and *e*, respectively. Thus, the time complexity becomes O_ANN_(*d*_*t*_ × *e* × (*i*. *h* + *h*. *o*)). In ACO, with *p* as the dimension of the given problem and *a* as the number of ants in *t* as iteration, the computational complexity is O_ACO_(*t* × *p*^*2*^ × *a*). In BBO tasks, the time complexity depends on the migration and mutation i.e. O_BBO_(g(migration)) + O(mutation), where g is the maximum number of generations. In the worst case, O_migration_(*f* × *s*^*2*^) where *f* and *s* are the number of features (i.e., SIVs) and solutions (i.e., habitats) and O_mutation_ (*f* × *s*). Therefore, it will be O_BBO_(*f* × *s*^*2*^ + *f* × *s*). Consequently, the computational complexity of the proposed models during execution can be calculated through the individual complexities of all models until all features are extended to infinity.

In a nutshell, the preparation steps before running the models include data segmentation, normalization, and initial settings. Also, statistical indicators will evaluate how to appraise the models’ performance, and cross-validation analysis will be considered to avoid overestimating the outcomes. Furthermore, the execution time analysis will be performed to check the efficiency of the models.

## Results and discussion

### Hyperparameter tuning of models

**Fig. 10:**
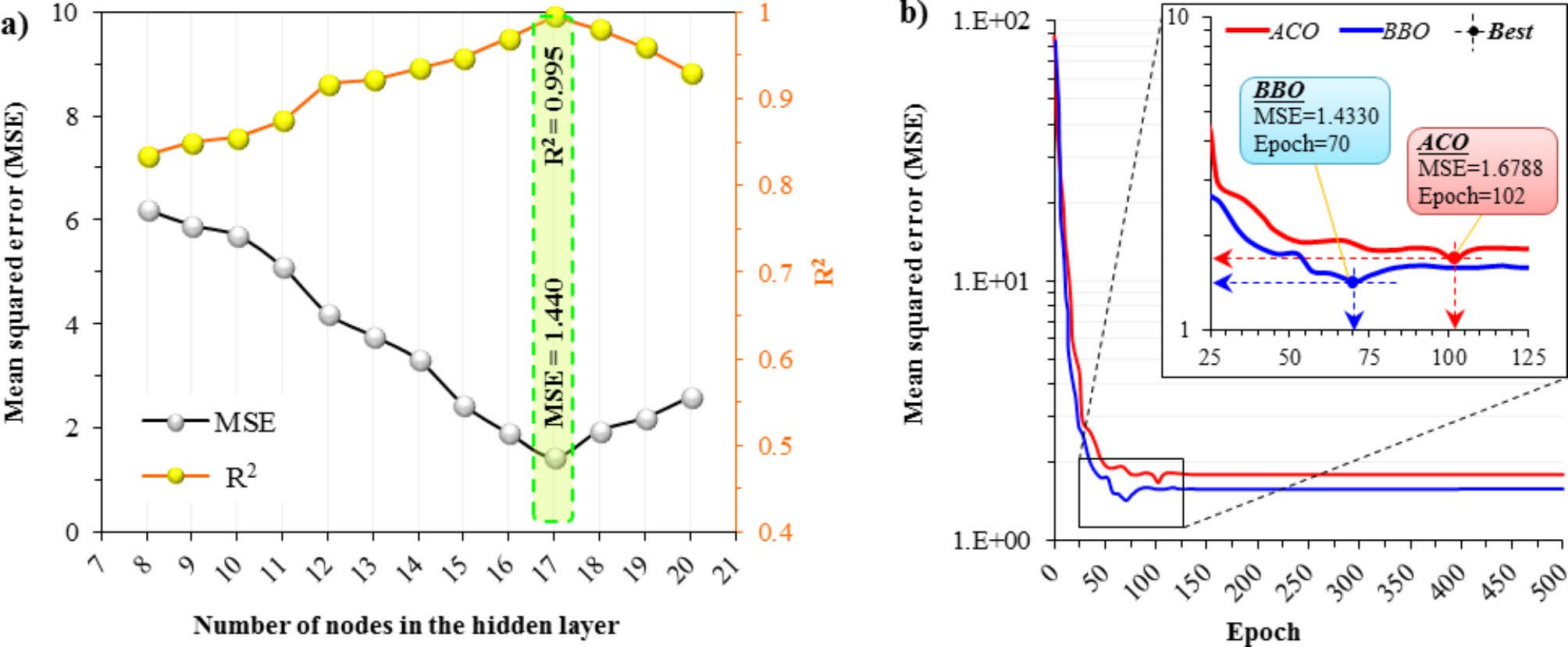
Comparative analysis of (**a**) No. of hidden nodes in ANN and (**b**) convergence curve of combination methods.

To finalize the architectural structure of the *7*-*H*_*nodes*_-*1* model (that is, determine *H*_*nodes*_), the model with a different number of nodes in the hidden layer was analyzed with the aim of minimizing the performance error. The models have been run on an identical dataset for an equitable comparison. Figure 10a shows the performance of different architectures by comparing MSE and R^2^ values. It can be observed that the architecture with *17* nodes in the hidden layer outperformed other numbers of nodes, as indicated by its lowest MSE = 1.440 and highest R^2^ = 0.995 values. Hence, the most suitable arrangement was considered *7*-*17*-*1* as the final architectural structure. Figure 10b displays the convergence performance of two hybrid methods used in this study. As can be seen, the best performance for ACO was 1.6788 at 102 epochs and for BBO was 1.4330 at 70 epochs. This indicates that the BBO method outperformed the ACO method in achieving a lower MSE in a lower number of epochs.

### Performance of the proposed models

**Fig. 11:**
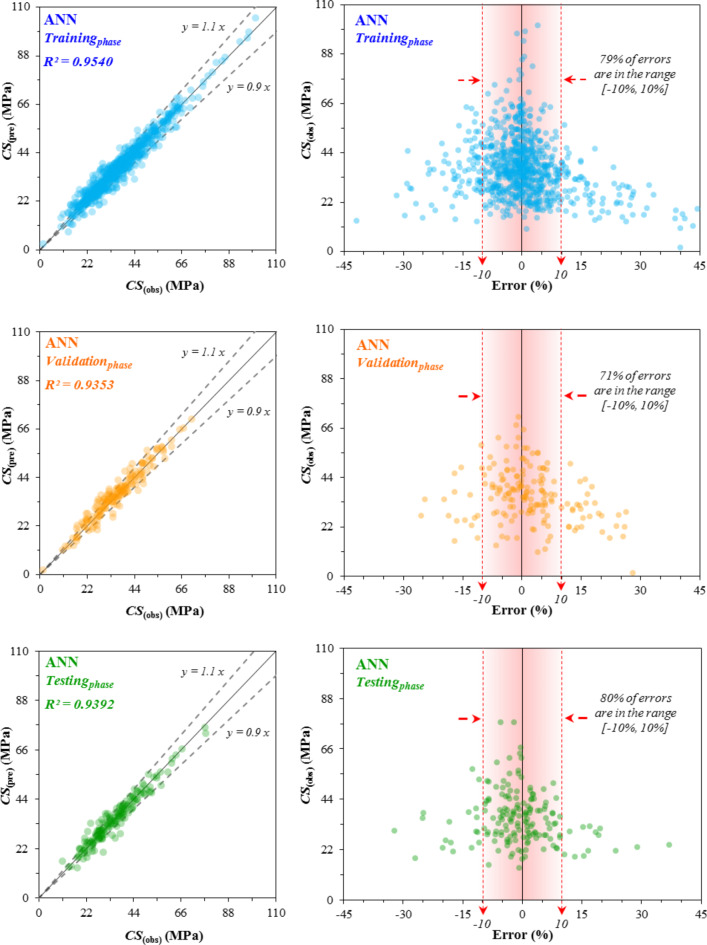
The observed vs. predicted ANN values of the CS, along with the error distribution.

**Fig. 12:**
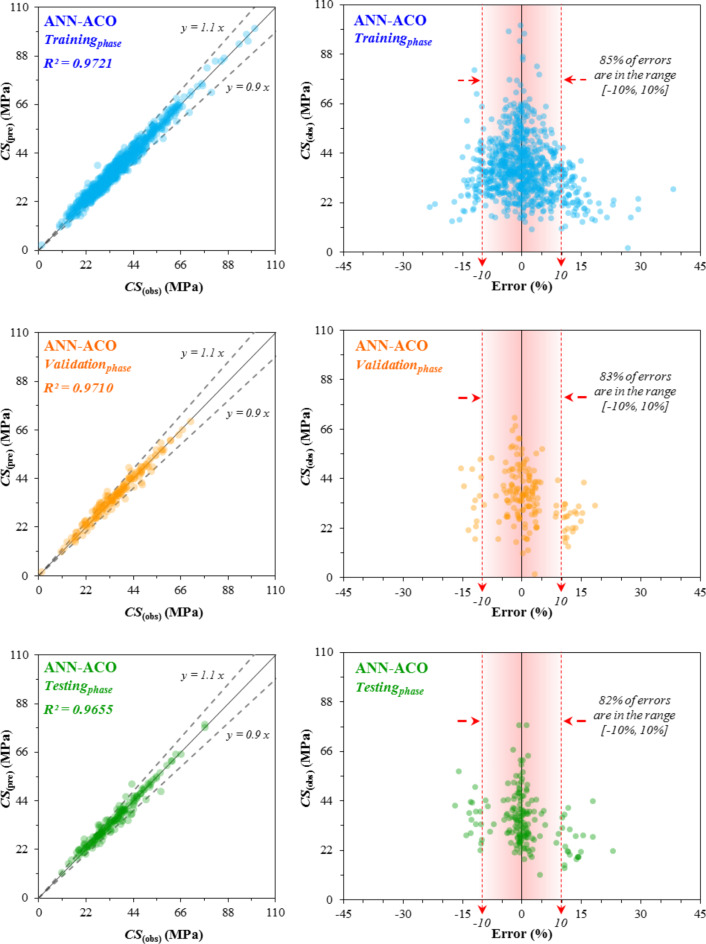
The observed vs. predicted ANN-ACO values of the CS, along with the error distribution.

**Fig. 13:**
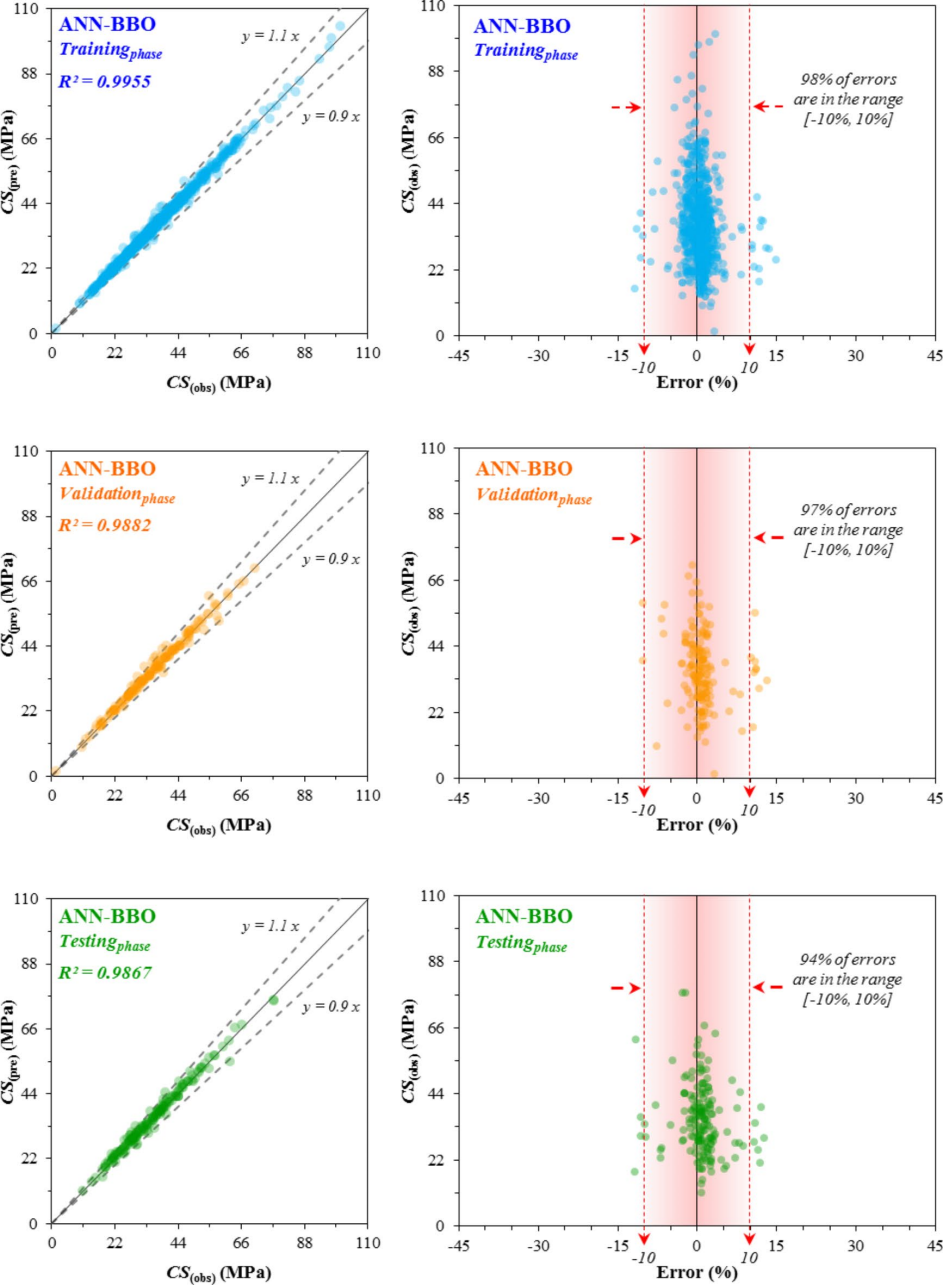
The observed vs. predicted ANN-BBO values of the CS, along with the error distribution.

The correlation between the predicted CS_(pre)_ values and the observed CS_(obs)_ values and the error distribution diagram across the training_*phase*_, validation_*phase*_, and testing_*phase*_ for the three developed models-ANN, ANN-ACO, and ANN-BBO are respectively illustrated in Figs. 11, 12 and 13. The R^2^-value achieved by the ANN model in the three phases (training_*phase*_, validation_*phase*_, and testing_*phase*_) was (0.9540, 0.9353, and 0.9392) while these values for the ANN-ACO and ANN-BBO models were (0.9721, 0.9710 and 0.9655) and (0.9955, 0.9882 and 0.9867), respectively. The results show a higher correlation between the observed and predicted values, such that the recorded data in the ANN-BBO model are closer to the best-fitting regression line (*y* = *x*) than the other two models. For further evaluation, the error distributions of each model in all three performance phases are shown in Figs. 11, 12 and 13. Upon closer examination of the error ranges, it was found that for the ANN-BBO model, 98%, 97%, and 94% of predictions in the training_*phase*_, validation_*phase*_, and testing_*phase*_, respectively, had errors within the range of [-10%, 10%]. In contrast, for the ANN-ACO and ANN models, the corresponding percentages were (85%, 83%, and 82%) and (79%, 71%, and 80%), respectively, which further confirms the better performance of the ANN-BBO model in predicting the CS of WMC in a narrower error range.

**Fig. 14:**
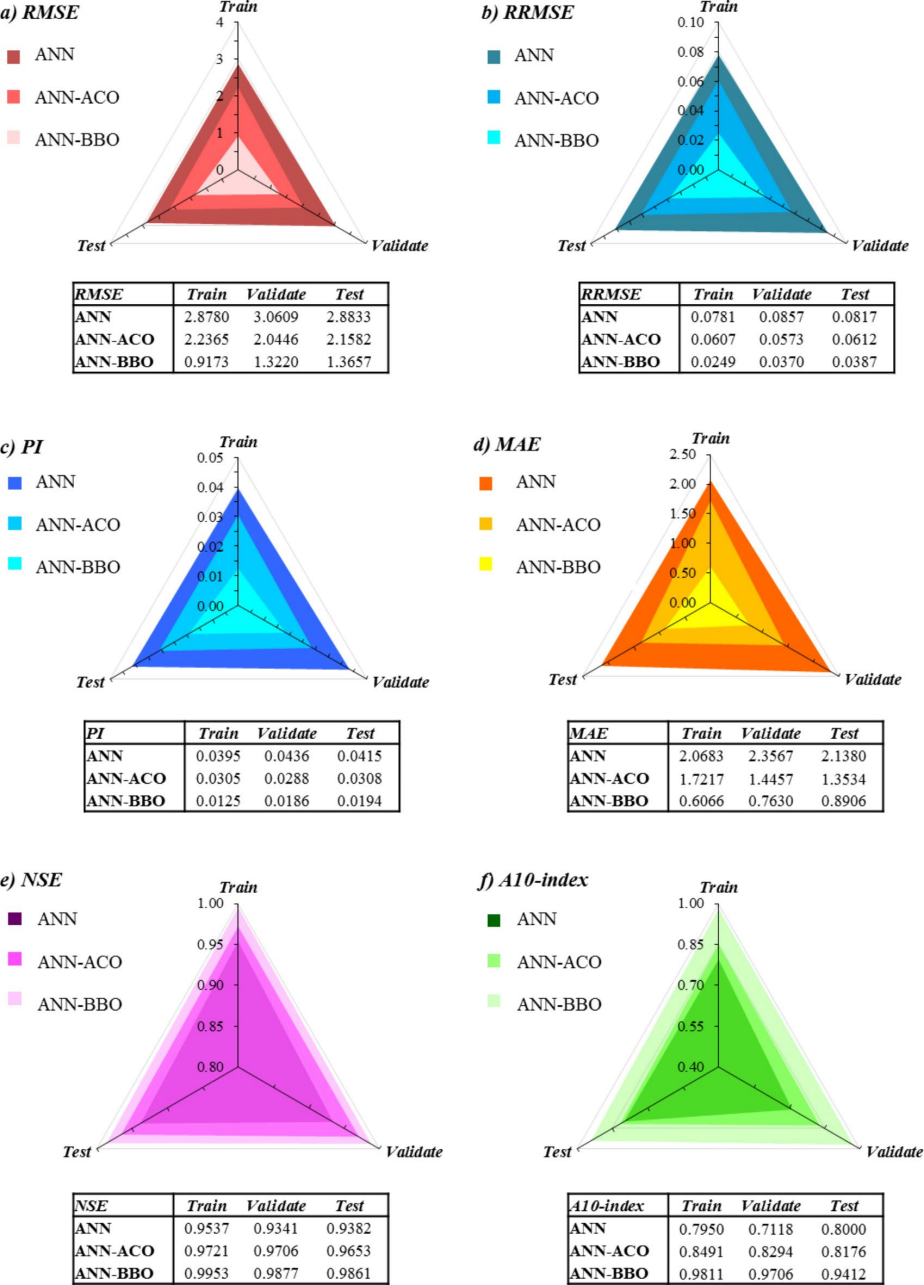
Comparative plots of the evaluation indicators for the developed models.

In Fig. 14, radar plots are used to visualize the sensitivity analysis of the three proposed models using six statistical measures of RMSE, RRMSE, PI, MAE, NSE, and A10-index. For better understanding, it is worth noting that the best performance for RMSE, RRMSE, PI, and MAE (Fig. 14a–d) was close to zero, while the best responses for NSE and A10-index (Fig. 14e, f) were close to one. According to Fig. 14, the ANN-BBO model exhibits less error in all four RMSE, RRMSE, PI, and MAE indicators. For example, these findings highlight that the ANN-BBO model outperformed the ANN and ANN-ACO models by a decrease of about 68% and 59%, respectively, compared to those models for RMSE, RRMSE, PI, and MAE in the training_*phase*_ (compare Fig. 14a–d). Also, the NSE index shows better performance of the ANN-BBO model than other models. In Fig. 14f, the A10-index evaluates the percentage of the data records that meet the condition of 0.9 < CS_obs_ / CS_pre_ < 1.1, so that a higher A10-index infers greater reliability of the proposed model. The results demonstrate that the ANN-BBO model is associated with higher A10-index values compared to the other two models for training_*phase*_, validation_*phase*_, and testing_*phase*_. A summary of the results is presented in Table 5. The results of statistical indicators align with earlier results regarding the effectiveness of the ANN-BBO model in predicting the CS of WMC.

**Table 5:** Comparison of evaluation indicators. [^*^It represents the accuracy of the prediction according to the range in Table 4].

Model	Phase	Indicators						
		*R*	RMSE (MPa)	MAE (MPa)	PI	NSE	A10-index	RRMSE
ANN	Training_*phase*_	0.9767	2.8780	2.0683	0.0395	0.9537	0.7950	0.0781_Good_^*^
	Validation_*phase*_	0.9671	3.0609	2.3567	0.0436	0.9341	0.7118	0.0857_Good_
	Testing_*phase*_	0.9691	2.8833	2.1380	0.0415	0.9382	0.8000	0.0817_Good_
ANN-ACO	Training_*phase*_	0.9859	2.2365	1.7217	0.0305	0.9721	0.8491	0.0607_Good_
	Validation_*phase*_	0.9854	2.0446	1.4457	0.0288	0.9706	0.8294	0.0573_Good_
	Testing_*phase*_	0.9826	2.1582	1.3534	0.0308	0.9653	0.8176	0.0612_Good_
ANN-BBO	Training_*phase*_	0.9977	0.9173	0.6066	0.0125	0.9953	0.7950	0.0249_Excellent_
	Validation_*phase*_	0.9941	1.3220	0.7630	0.0186	0.9877	0.7118	0.0370_Excellent_
	Testing_*phase*_	0.9933	1.3657	0.8906	0.0194	0.9861	0.8000	0.0387_Excellent_

**Fig. 15:**
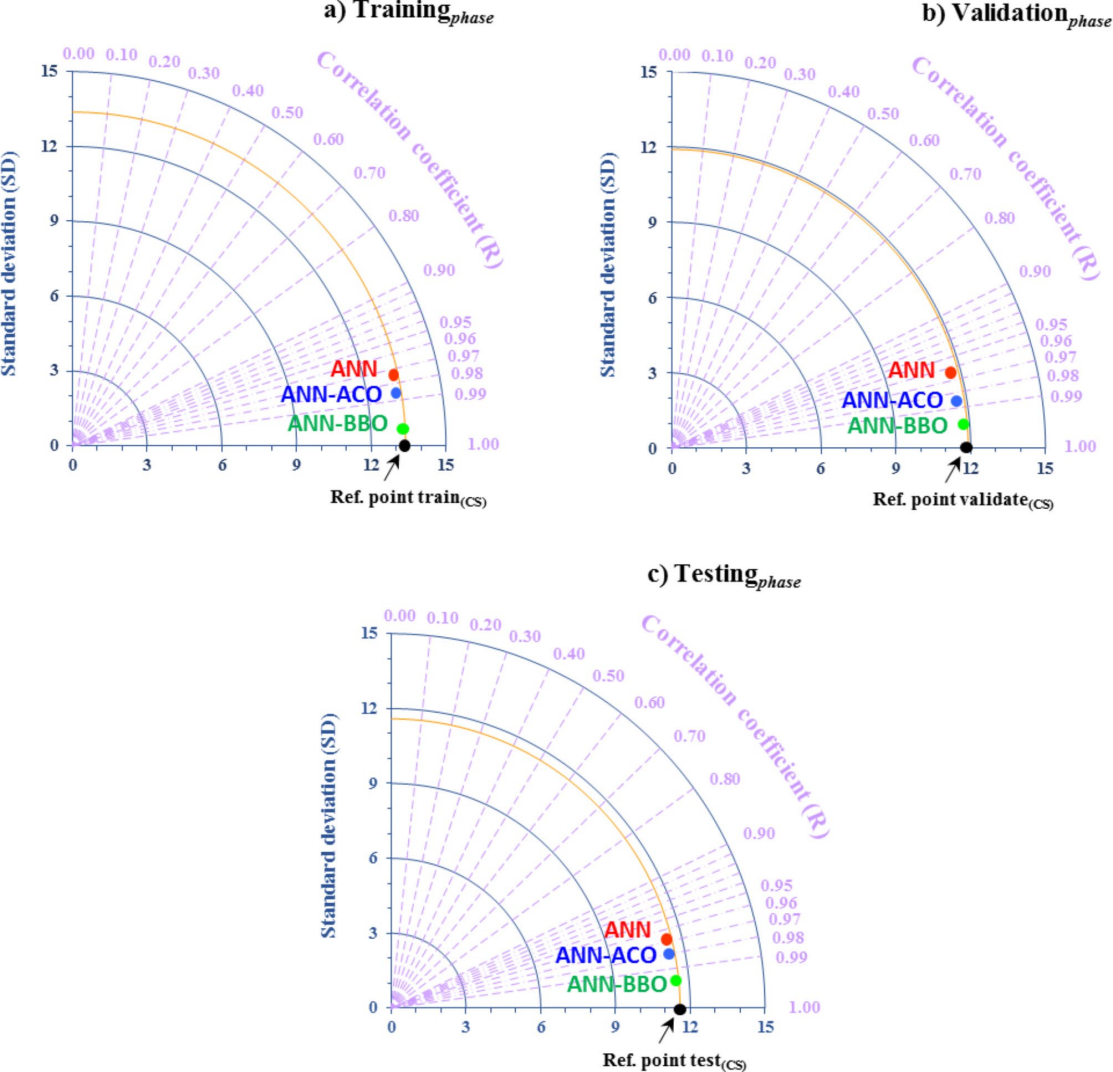
Taylor diagrams of the developed models for (**a**) Training_*phase*_, (**b**) Validation_*phase*_, and (**c**) Testing_*phase*_.

Besides, to better reveal the performance of the developed models, two evaluation indicators-standard deviation (SD) and correlation coefficient (R) are integrated into polar coordinates. The resulting diagram is known as a Taylor diagram, which represents the compliance between the predicted and observed values^115^. The best performance of the model is achieved when it is closer to the reference point (Ref. point). As can be observed in Fig. 15, all three models show a strong correlation with the Ref. point. However the ANN-BBO model is nearer to the Ref. point in the training_*phase*_, validation_*phase*_, and testing_*phase*_. This outcome proves that the ANN-BBO model can be a more robust and efficient model than the ANN and ANN-ACO models.

**Fig. 16:**
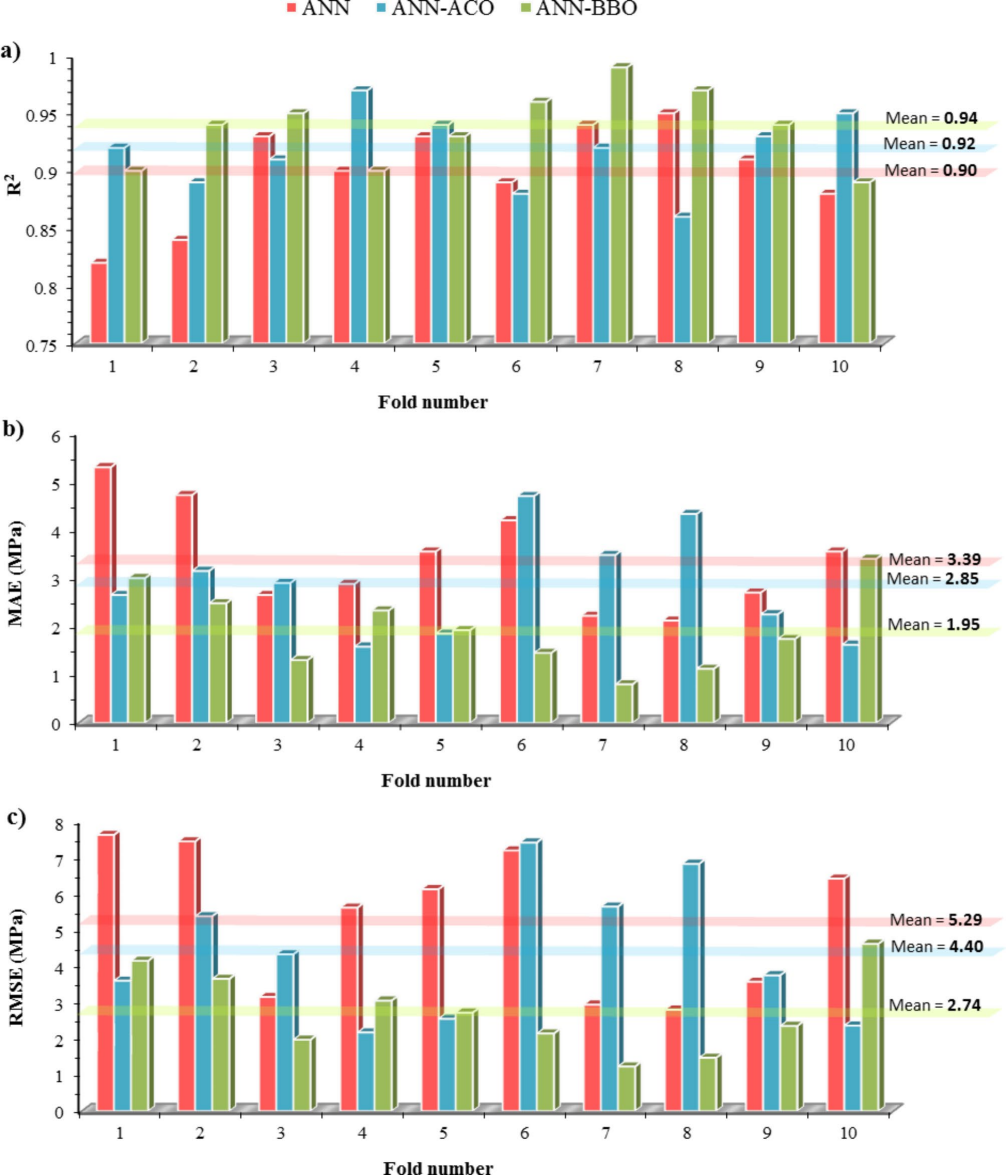
(**a**) R^2^, (**b**) MAE, and (**c**) RMSE values for 10-fold cross-validation analysis of the developed models.

*K*-fold cross-validation was undertaken to focus more on the validity and robustness of the developed models and to examine their reliability and ability to handle new data. This comprehensive analysis was repeated through a process ten times and evaluated using statistical indicators of R^2^, MAE, and RMSE, as shown in Fig. 16. The mean performance of R^2^ values for the models of ANN, ANN-ACO, and ANN-BBO is 0.90, 0.92, and 0.94, respectively. In addition, the ANN-BBO model experienced a mean MAE of 1.95, whereas it had an error reduction of 43% and 32%, respectively, compared to the mean MAE of the ANN (3.39) and ANN-ACO (2.85) models. A similar decreasing trend is also observed in the mean RMSE value. Besides, the computational time of the models indicates that BBO took a shorter time, with a mean of 0.86 s, while ACO has a mean of 1.02 s. As a consequence, the BBO technique has a faster execution time compared to the ACO technique. From the findings of this analysis, it can be concluded that the proposed ANN-BBO model possesses superior performance capability. Table 6 presents the overall results of the 10-fold analysis for the three developed models.

**Table 6:** Analysis of 10-fold cross-validation along with the computational time for the proposed models.

10-Fold	ANN					ANN-ACO					ANN-BBO			
	R^2^	MAE (MPa)	RMSE (MPa)	Time (sec)		R^2^	MAE (MPa)	RMSE (MPa)	Time (sec)		R^2^	MAE (MPa)	RMSE (MPa)	Time (sec)
1	0.82	5.30	7.63	1.3		0.92	2.65	3.60	1.2		0.90	3.00	4.15	0.9
2	0.84	4.72	7.45	1.2		0.89	3.15	5.38	1.1		0.94	2.48	3.65	0.8
3	0.93	2.65	3.15	1.0		0.91	2.90	4.33	1.0		0.95	1.30	1.97	0.8
4	0.90	2.88	5.62	1.0		0.97	1.58	2.17	1.0		0.90	2.33	3.05	0.9
5	0.93	3.55	6.13	1.0		0.94	1.85	2.55	1.0		0.93	1.92	2.72	0.9
6	0.89	4.20	7.20	1.2		0.88	4.70	7.42	1.1		0.96	1.45	2.14	0.9
7	0.94	2.22	2.94	1.0		0.92	3.48	5.65	0.9		0.99	0.80	1.23	0.8
8	0.95	2.12	2.80	1.0		0.86	4.33	6.83	0.9		0.97	1.12	1.47	0.8
9	0.91	2.70	3.57	1.1		0.93	2.25	3.75	0.9		0.94	1.74	2.35	0.9
10	0.88	3.55	6.42	1.2		0.95	1.62	2.36	1.1		0.89	3.40	4.62	0.9
Min	0.82	2.12	2.80	1.0		0.86	1.58	2.17	0.9		0.89	0.80	1.23	0.8
Max	0.95	5.30	7.63	1.3		0.97	4.70	7.42	1.2		0.99	3.40	4.62	0.9
Mean	0.90	3.39	5.29	1.1		0.92	2.85	4.40	1.02		0.94	1.95	2.74	0.86

### Contribution of input variables

**Fig. 17:**
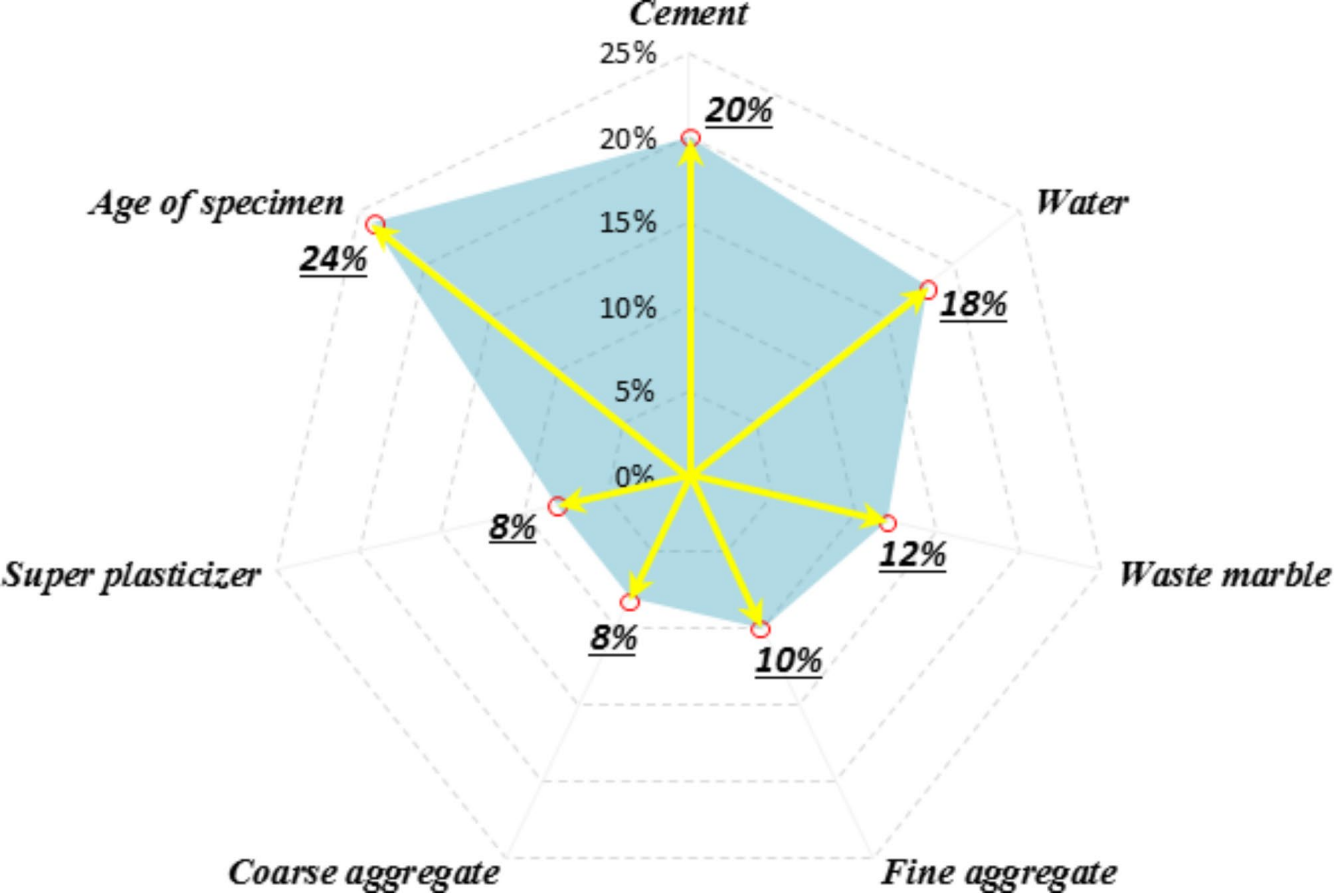
The contribution of input variables in the best-proposed model on the CS of WMC.

A comparative analysis was performed to elucidate the effectiveness of each input variable in the best model’s performance in predicting the CS of WMC. For this purpose, the importance of the inputs is specified by evaluating the influence on the model performance when each one is removed and not employed by the model. This approach enables the identification of input variables whose exclusion would significantly reduce the model performance, indicating their pivotal role in the prediction process. Figure 17 depicts the significance of the inputs taken into account in the best model (ANN-BBO). Notably, all inputs participate in model performance but with varying levels of importance. As illustrated in Fig. 17, the specimen’s age is the main influencing variable in the CS of WMC, with a contribution of 24%, consistent with previous studies’ findings^44,47^. Following that, cement (20%), water (18%), and waste marble (12%), respectively, are the next three important variables influencing the prediction of the CS in WMC. It reveals that after cement and water as the fundamental ingredients in cementitious material, WM plays the largest contribution in sustainable concrete mixtures containing WM due to its role as an alternative to cement. The remaining input variables, 10%, 8%, and 8%, belong to the variables of fine aggregate, coarse aggregate, and superplasticizer, respectively.

**Fig. 18:**
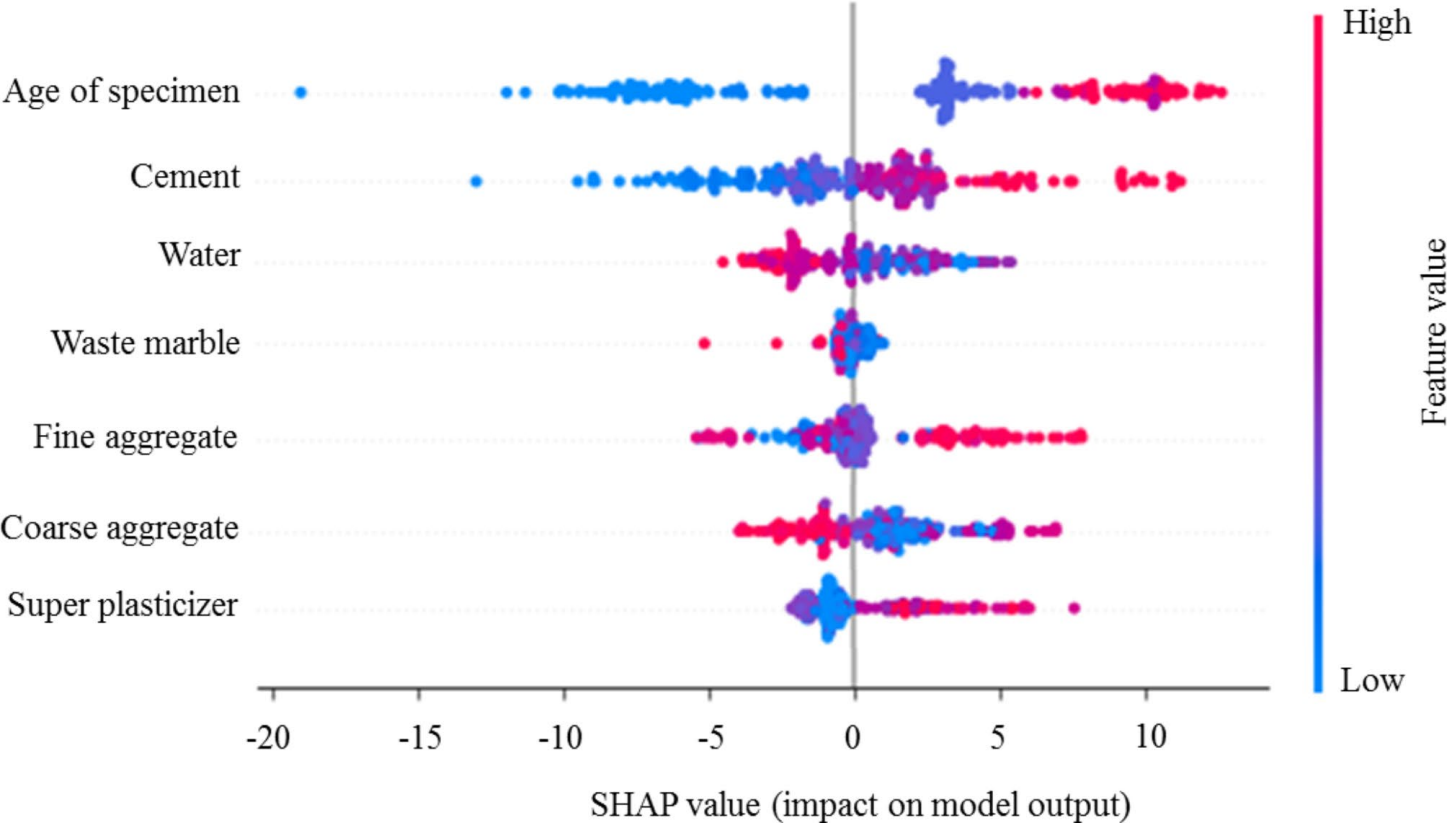
Positive-negative effects of input variables on the CS of WMC using SHAP analysis.

To further evaluate the impact of inputs, SHAP analysis^116^, rooted in game theory, is established to interpret the impact of each input variable on the model output. Figure 18 depicts the significance of each variable by illustrating its impact on prediction accuracy. The color spectrum denotes the value of each variable, ranging from low (soft blue) to high (vibrant red). Each point on the summary plot corresponds to an individual observation from the compiled dataset. The horizontal axis of the plot shows the positive or negative impact of each data point on the prediction accuracy of the model output. A positive impact indicates that the selected sample boosts prediction accuracy, while a negative impact implies the opposite. Figure 18 shows the widest distribution of the variables among the specimen ages, indicating its significant impact on predictions. The figure also illustrates that, as expected, high values of age and cement (depicted in red) positively impact the model’s accuracy in predicting CS of WMC, while this positive impact is achieved in low values for water and waste marble (depicted in blue).

**Fig. 19:**
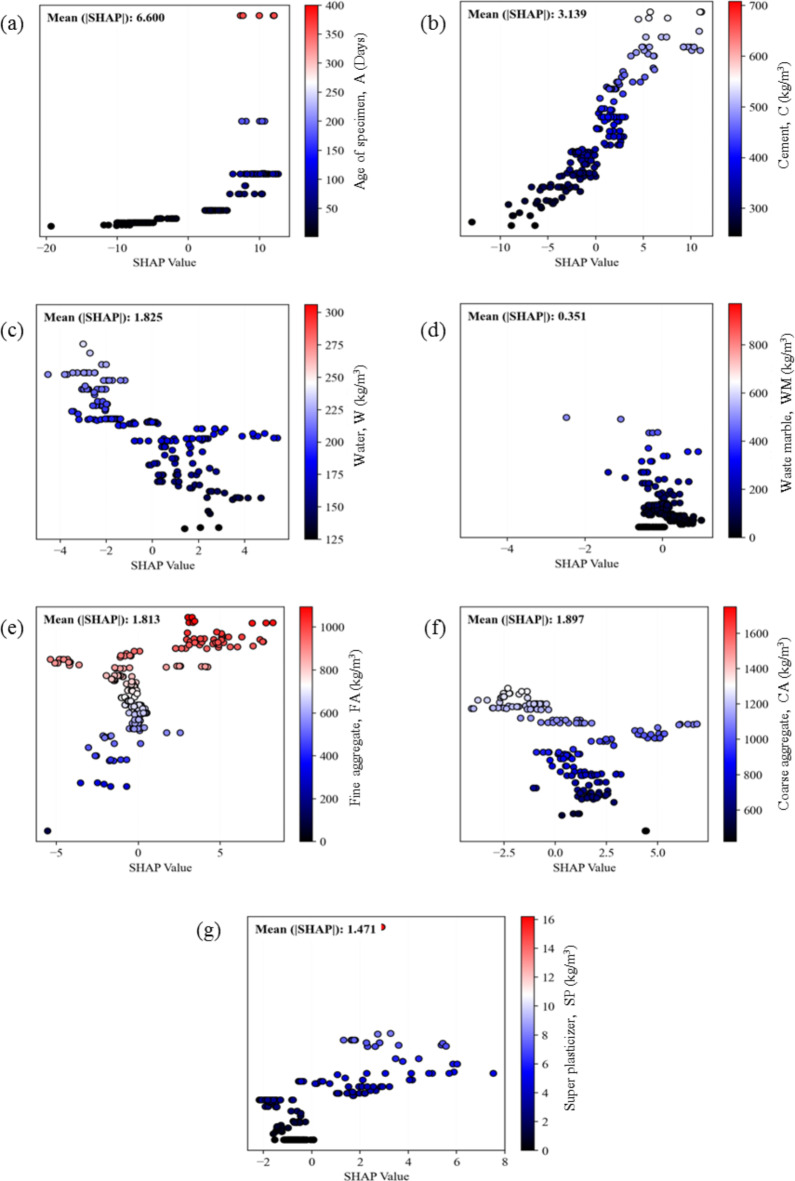
Summary plots of SHAP analysis regarding the effects of (**a**) age, (**b**) cement, (**c**) water, (**d**) waste marble, (**e**) fine aggregate, (**f**) coarse aggregate, and (**g**) super plasticizer.

The SHAP global interpretation of the ANN-BBO model provides a comprehensive visualization conducive to identifying underlying data trends, as shown in Fig. 19. Each point along the horizontal axis corresponds to Shapley values specific to individual input variables per prediction, with color coding representing the respective input variable values. In summary, certain variables positively impact CS, including higher values of age and cement, as shown in Fig. 19a and b. Specifically, SHAP values for the age of the specimen exhibit a significant range from − 20 to 15, underscoring its substantial impact on CS, thereby indicating that greater ages are advantageous for achieving higher CS. Regarding the effect of C, mass values of more than 350 kg/m^3^ have resulted in positive SHAP values, which means a positive effect on CS. On the contrary, increasing water values ​​negatively affects CS, as can be seen from Fig. 19c. The water contents with SHAP values ranging from 0 to 4 show the potential for higher CS. For the effect of WM, the SHAP values for weights lower than 200 kg/m^3^ have a high density between 0 and 1. However, by increasing above this amount, the SHAP values expand in the negative direction of the x-axis (see Fig. 19d). This indicates that the optimum substitution for waste marble can be up to 15% of the total cement content. Also, SHAP analysis illustrates the positive effect of FA on CS in the SHAP values of 0 to 5 in the amount of 800–1000 kg/m^3^ (Fig. 19e). Furthermore, it can be seen that the distribution of data points does not show a clear pattern for the amount of CA and SP, indicating their minimal effect on strength (Fig. 19f and g). Accordingly, this analysis is helpful since it sheds light on the positive or negative impact of each input variable on the model output and provides in-depth insight into the role of variables and their effect on the model predictions.

### Comparing the current best model with the literature

To further validate the best-proposed model (ANN-BBO) in the present study, six recent studies on predicting the CS of WMC with different predictive models, including XGB^44^, RF^45^, RT^35^, ANFIS^46^, GP^47^, and ANN^48^, have been considered. Table 7 evaluates the accuracy of the trained models by gathering their performance results from three statistical indicators-R, MAE, and RMSE and adopting a comparison between them. Comparing the results indicates that the ANN-BBO model outperformed the best-proposed models in the literature in terms of providing a higher correlation coefficient (R) and lower error (MAE and RMSE). The evaluation of the literature shows that the highest accuracy belongs to the models with the least number of data; for example, R-value = 0.9902 for^48^ with 49 data points and R-value = 0.9972 for^46^ with 130 data points. This means that achieving these models with high accuracy cannot indicate their strong performance, while their performance against a wider database is still questionable. In contrast, the present study performs better on a larger dataset than previous studies, proving the proposed model’s superiority and inclusiveness. Therefore, the performed comparison strongly confirms the improved accuracy and reliability of the hybrid ANN-BBO model compared to the previous single models in predicting the CS of WMC.

**Table 7:** Comparing the current best model with literature.

Authors (Year) [Ref.]	No. data used	The best-proposed model	Statistical indicators		
			*R* achieved	MAE achieved (MPa)	RMSE achieved (MPa)
Current study	1135	ANN-BBO_Different ages_	0.9977	0.6066	0.9173
		ANN-BBO_28 − day_	0.9930	0.7342	1.2510
For compressive strength with different ages					
Shamsabadi et al. (2023)^44^	630	XGB	0.9899	1.4900	2.1500
Sharma et al. (2023)^35^	158	RT	0.9773	0.8776	1.5914
Sharma et al. (2023)^46^	130	ANFIS	0.9972	0.9587	1.4916
Sharma et al. (2022)^47^	189	GP	0.9704	1.2800	1.8734
For 28-day compressive strength					
Singh et al. (2022)^46^	720	RF	0.9622	1.6080	9.6790
Sharma et al. (2022)^48^	49	ANN	0.9902	-	1.7941

## Conclusions and future directions

WM as an industrial by-product can effectively be utilized to replace both cement and fine aggregate in concrete, which has resulted in several environmental and economic benefits, including the reduction of (i) disposal problems and its costs, (ii) natural resource extraction, (iii) energy consumption, and (iv) environmental pollution. Evaluating the effectiveness of WM on the CS of concrete is significant before its widespread incorporation into concrete mixes, given the importance of CS as a design criterion for concrete structures. Considering that the nature of laboratory activities is time-consuming and expensive and requires re-testing for changes in the ratio of materials and conditions, this study seeks to provide an accurate and reliable AI-driven approach for predicting the CS of WMC. Hence, two metaheuristic algorithms-ACO and BBO were proposed as robust hybrid optimization techniques to outperform the single ANN model. After evaluating the developed models, the best-proposed model was compared with literature models. A concise summary of the findings is as follows:

- Evaluating all statistical indicators indicated that the ANN-BBO model outperformed the other two models of single ANN and ANN-ACO, with R^2^ values of 0.9955, 0.9882, and 0.9862 for the training_*phase*_, validation_*phase*_, and testing_*phase*_, respectively.
- The error distribution of the models revealed that 98% of the predicted data points in the training_*phase*_ by the ANN-BBO model experienced errors in the range of [-10%, 10%], whereas for the ANN-ACO and ANN models, this percentage was 85% and 79%, respectively. It indicated that the ANN-BBO model can provide a more accurate model with a narrower error range compared to the other models.
- The 10-fold cross-validation analysis demonstrated the reliability and generalizability of the ANN-BBO model using statistical indicators of R^2^, MAE, and RMSE.
- Analyzing the importance of input variables showed that the most influential variable controlling the CS of WMC was the specimen’s age, with a contribution of 24%, followed by cement (20%), water (18%), and WM (12%).
- The SHAP analysis identified that the high values of age and cement positively impact the CS of WMC, while this trend for water and waste marble is negative. According to the SHAP summary plot, the optimum substitution for waste marble suggests up to 15% of the total cement content.
- Eventually, to validate the ANN-BBO model, a comparison was performed with the results of previous studies’ models. Findings revealed that the proposed model provides by far the best performance in predicting the CS of WMC compared to the literature models. Furthermore, it was developed on a larger dataset than previous studies, confirming its superiority.

Future research directions could include: (i) adopting different additional variables and evaluating the durability and strength of WMC under different environmental conditions; and (ii) developing new AI models to achieve a more reliable model by expanding a more comprehensive database and utilizing new metaheuristic optimization techniques.

## Electronic Supplementary Material

Below is the link to the electronic supplementary material.

Supplementary Material 1

## Data Availability

The database used in this study is available in the supplementary file.

## Abbreviations

***A***: Age of specimens
***A10***: A10-index
***ACO***: Ant colony optimization
***AdB***: AdaBoost
***AI***: Artificial intelligence
***ANFIS***: Adaptive neuro-fuzzy inference system
***ANN***: Artificial neural network
***BBO***: Biogeography-based optimization
***Bg***: Bagging
***CA***: Coarse aggregate
***CS***: Compressive strength
${{\varvec{C}}{\varvec{S}}}_{{\varvec{i}},\boldsymbol{ }{\varvec{o}}{\varvec{b}}{\varvec{s}}}$: Observed *C**S* of the *i*th data
${{\varvec{C}}{\varvec{S}}}_{{\varvec{i}},\boldsymbol{ }{\varvec{p}}{\varvec{r}}{\varvec{e}}}$: Predicted *C**S* of the *i*th data
$\overline{{{\varvec{C}}{\varvec{S}} }_{{\varvec{o}}{\varvec{b}}{\varvec{s}}}}$: Average of the observed *CS*
$\overline{{{\varvec{C}}{\varvec{S}} }_{{\varvec{p}}{\varvec{r}}{\varvec{e}}}}$: Average of the predicted *CS*
***DT***: Decision tree
***FA***: Fine aggregate
***GB***: Gradient boosting
***GP***: Gaussian processes
***H***: Number of neurons in the hidden layer
***HSI***: Habitat suitability index
***KNN***: K-nearest neighbors
***LSSVR***: Least-square support vector regression
***m10***: Number of data in which *CS*_*ob*__s_/*CS*_*pre*_ fits in the range of 0.90–1.10
***MAE***: Mean absolute error
***MARS***: Multivariate adaptive regression spline
***MEP***: Multi expression programming
***MLP***: Multilayer perceptron neural network
***MPA***: Marine predators algorithm
***NSE***: Nash–Sutcliffe efficiency
***PI***: Performance index
***PSO***: Particle swarm optimization
***R***: Correlation coefficient
***R***^***2***^: Coefficient of determination
***RBF***: Radial basis function
***RF***: Random forest
***RMSE***: Root mean squared error
***RRMSE***: Relative root mean squared error
***RT***: Random tree
***SD***: Standard deviation
***SHAP***: Shapley additive explanations
***SIV***: Suitable index variables
***SP***: Superplasticizer
***SVM***: Support vector machine
***SVR***: Support vector regression
***T***: Total of data
***W***: Water
***WM***: Waste marble
***WMC***: Waste marble concrete
***XGB***: Extreme gradient boosting
